# Supplementary material for: Multi-omic underpinnings of epigenetic aging and human longevity
Source: Nat Commun. 2023 Apr 19;14:2236. doi: 10.1038/s41467-023-37729-w (PMC10115892; doi:10.1038/s41467-023-37729-w)
Supplement: Supplementary file 1 — Supplementary Information [file 41467_2023_37729_MOESM1_ESM.pdf]

# Multi-omic underpinnings of epigenetic aging and human longevity

Lucas A. Mavromatis[1#], ScB; Daniel B. Rosoff[1,2#], AB, ScB; Andrew S. Bell[1], BA; Jeesun Jung[1], PhD; Josephin Wagner[1], MD, MSc; Falk W. Lohoff[1*], MD.

[1]Section on Clinical Genomics and Experimental Therapeutics, National Institute on Alcohol Abuse and Alcoholism, National Institutes of Health, Bethesda, MD, USA

[2]NIH-Oxford-Cambridge Scholars Program; University of Oxford, UK

***Corresponding Author:**

Falk W. Lohoff, M.D.

Chief, Section on Clinical Genomics and Experimental Therapeutics (CGET)

Lasker Clinical Research Scholar

National Institute on Alcohol Abuse and Alcoholism (NIAAA)

National Institutes of Health

10 Center Drive (10CRC/2-2352)

Bethesda, MD 20892-1540

Office: 301-827-1542

falk.lohoff@nih.gov

**[#]These authors contributed equally**

**Supplementary Note 1 (STROBE-MR Checklist)**

1. TITLE and ABSTRACT

*Indicate Mendelian randomization as the study's design in the title and/or the abstract.*

Abstract

INTRODUCTION

2. Background

*Explain the scientific background and rationale for the reported study. Is causality between exposure and outcome plausible? Justify why MR is a helpful method to address the study question.*

Addressed in the Introduction and Methods

3. Objectives

*State specific objectives clearly, including pre-specified causal hypotheses (if any).*

Addressed in the Introduction.

METHODS

4. Study design and data sources

*Present key elements of study design early in the paper. Consider including a table listing sources of data for all phases of the study. For each data source contributing to the analysis, describe the following:*

*a) Describe the study design and the underlying population from which it was drawn. Describe also the setting, locations, and relevant dates, including periods of recruitment, exposure, follow-up, and data collection, if available.*

*b) Give the eligibility criteria, and the sources and methods of selection of participants.*

*c) Explain how the analyzed sample size was arrived at.*

*d) Describe measurement, quality and selection of genetic variants.*

*e) For each exposure, outcome and other relevant variables, describe methods of assessment and, in the case of diseases, the diagnostic criteria used.*

*f) Provide details of ethics committee approval and participant informed consent, if relevant.*

Addressed in the Methods.

5. Assumptions

*Explicitly state assumptions for the main analysis (e.g. relevance, exclusion, independence, homogeneity) as well assumptions for any additional or sensitivity analysis.*

Addressed in the Methods.

6. Statistical methods: main analysis

*Describe statistical methods and statistics used.*

*a) Describe how quantitative variables were handled in the analyses (i.e., scale, units, model).*

*b) Describe the process for identifying genetic variants and weights to be included in the analyses (i.e, independence and model). Consider a flow diagram.*

*c) Describe the MR estimator, e.g. two-stage least squares, Wald ratio, and related statistics.*

*Detail the included covariates and, in case of two-sample MR, whether the same covariate set was used for adjustment in the two samples.*

*d) Explain how missing data were addressed.*

*e) If applicable, say how multiple testing was dealt with.*

Addressed in the Methods.

7. Assessment of assumptions

*Describe any methods used to assess the assumptions or justify their validity.*

Addressed in the Methods.

8. Sensitivity analyses

*Describe any sensitivity analyses or additional analyses performed.*

Addressed in the Methods.

9. Software and pre-registration

*a) Name statistical software and package(s), including version and settings used.*

Addressed in the Methods.

*b) State whether the study protocol and details were pre-registered (as well as when and where).*

Addressed in the Methods.

RESULTS

10. Descriptive data

*a) Report the numbers of individuals at each stage of included studies and reasons for exclusion. Consider use of a flow-diagram.*

*b) Report summary statistics for phenotypic exposure(s), outcome(s) and other relevant variables (e.g. means, standard deviations, proportions).*

*c) If the data sources include meta-analyses of previous studies, provide the number of studies, their reported ancestry, if available, and assessments of heterogeneity across these studies. Consider using a supplementary table for each data source.*

*d) For two-sample Mendelian randomization:*

*i. Provide information on the similarity of the genetic variant-exposure associations between the exposure and outcome samples.*

*ii. Provide information on extent of sample overlap between the exposure and outcome data sources.*

Addressed in the Methods, Results and Supplementary Data.

11. Main results

*a) Report the associations between genetic variant and exposure, and between genetic variant and outcome, preferably on an interpretable scale (e.g. comparing 25th and 75th percentile of allele count or genetic risk score, if individual-level data available).*

*b) Report causal effect estimate between exposure and outcome, and the measures of uncertainty from the MR analysis. Use an intuitive scale, such as odds ratio, or relative risk, per standard deviation difference.*

*c) If relevant, consider translating estimates of relative risk into absolute risk for a meaningful time-period.*

*d) Consider any plots to visualize results (e.g. forest plot, scatterplot of associations between genetic variants and outcome versus between genetic variants and exposure).*

Addressed in the Results and Supplementary Data.

12. Assessment of assumptions

*a) Assess the validity of the assumptions.*

*b) Report any additional statistics (e.g., assessments of heterogeneity, such as I2, Q statistic).*

Addressed in the Results, Supplementary Data and Discussion.

13. Sensitivity and additional analyses

a) *Use sensitivity analyses to assess the robustness of the main results to violations of the assumptions.*

*b) Report results from other sensitivity analyses (e.g., replication study with different dataset, analyses of subgroups, validation of instrument(s), simulations, etc.).*

*c) Report any assessment of direction of causality (e.g., bidirectional MR).*

*d) When relevant, report and compare with estimates from non-MR analyses.*

*e) Consider any additional plots to visualize results (e.g., leave-one-out analyses).*

Addressed in the Results and Supplementary Data.

DISCUSSION

14. Key results

*Summarize key results with reference to study objectives.*

Addressed in the Discussion.

15. Limitations

*Discuss limitations of the study, taking into account the validity of the MR assumptions, other sources of potential bias, and imprecision. Discuss both direction and magnitude of any potential bias, and any efforts to address them.*

Addressed in the Discussion.

16. Interpretation

*a) Give a cautious overall interpretation of results considering objectives and limitations.*

*Compare with results from other relevant studies.*

*b) Discuss underlying biological mechanisms that could be modelled by using the genetic variants to assess the relationship between the exposure and the outcome.*

*c) Discuss whether the results have clinical or policy relevance, and whether interventions could have the same size effect.*

Addressed in the Discussion.

17. Generalizability

*Discuss the generalizability of the study results (a) to other populations (i.e. external validity),*

*(b) across other exposure periods/timings, and (c) across other levels of exposure.*

Addressed in the Discussion.

OTHER INFORMATION

18. Funding

*Give the source of funding and the role of the funders for the present study and, if applicable, for the original study or studies on which the present article is based.*

Addressed in the Acknowledgements.

19. Data and data sharing

*Present data used to perform all analyses or report where and how the data can be accessed.*

*State whether statistical code is publicly accessible and if so, where.*

Addressed in the Methods.

20. Conflicts of Interest

*All authors should declare all potential conflicts of interest.*

Addressed in the Competing interests.

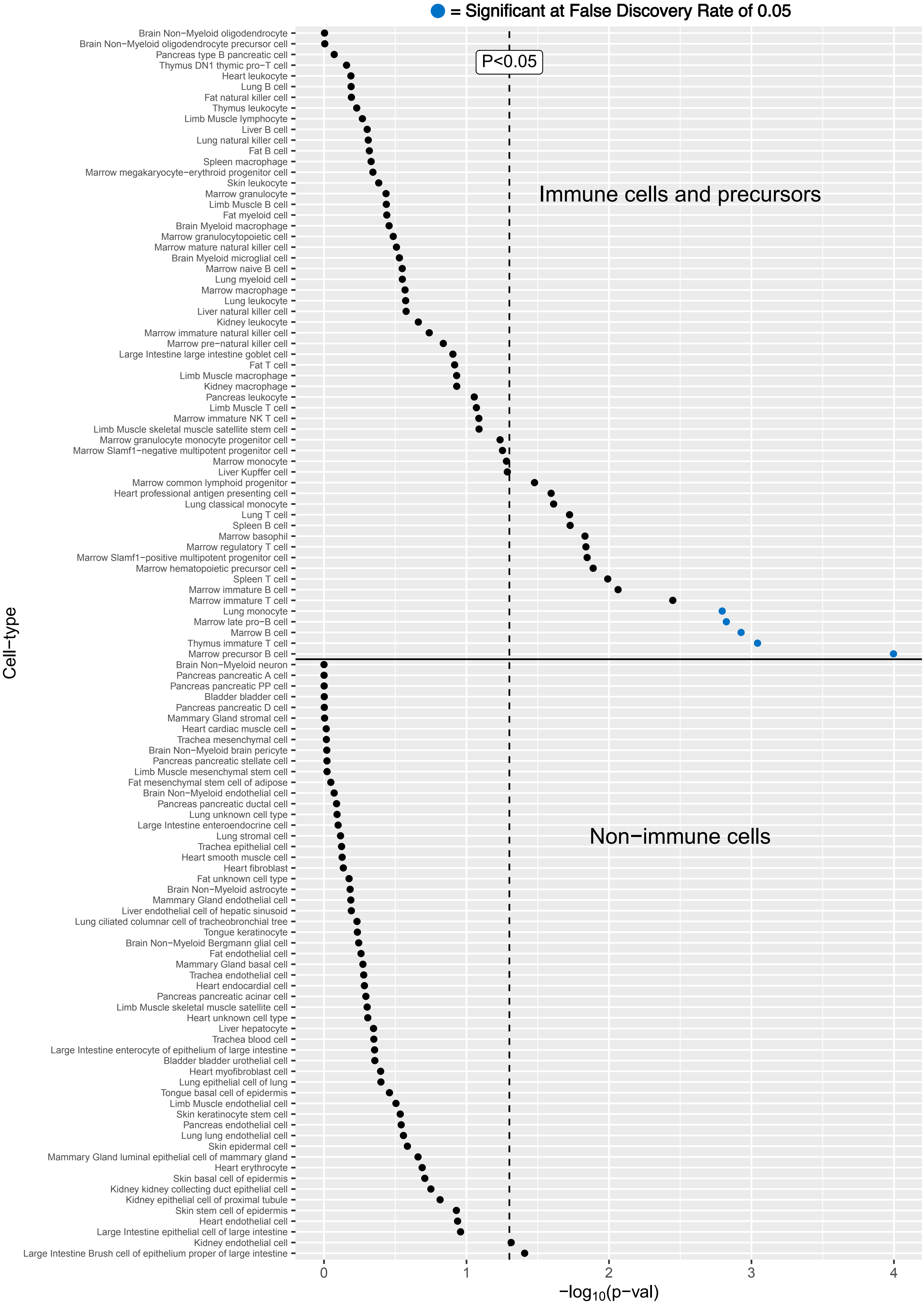
= Significant at False Discovery Rate of 0.05
P<0.05
Immune cells and precursors
Non−immune cells
Cell−type
−log10(p−val)
0
1
2
3
4
Brain Non−Myeloid oligodendrocyte
Brain Non−Myeloid oligodendrocyte precursor cell
Pancreas type B pancreatic cell
Thymus DN1 thymic pro−T cell
Heart leukocyte
Lung B cell
Fat natural killer cell
Thymus leukocyte
Limb Muscle lymphocyte
Liver B cell
Lung natural killer cell
Fat B cell
Spleen macrophage
Marrow megakaryocyte−erythroid progenitor cell
Skin leukocyte
Marrow granulocyte
Limb Muscle B cell
Fat myeloid cell
Brain Myeloid macrophage
Marrow granulocytopoietic cell
Marrow mature natural killer cell
Brain Myeloid microglial cell
Marrow naive B cell
Lung myeloid cell
Marrow macrophage
Lung leukocyte
Liver natural killer cell
Kidney leukocyte
Marrow immature natural killer cell
Marrow pre−natural killer cell
Large Intestine large intestine goblet cell
Fat T cell
Limb Muscle macrophage
Kidney macrophage
Pancreas leukocyte
Limb Muscle T cell
Marrow immature NK T cell
Limb Muscle skeletal muscle satellite stem cell
Marrow granulocyte monocyte progenitor cell
Marrow Slamf1−negative multipotent progenitor cell
Marrow monocyte
Liver Kupffer cell
Marrow common lymphoid progenitor
Heart professional antigen presenting cell
Lung classical monocyte
Lung T cell
Spleen B cell
Marrow basophil
Marrow regulatory T cell
Marrow Slamf1−positive multipotent progenitor cell
Marrow hematopoietic precursor cell
Spleen T cell
Marrow immature B cell
Marrow immature T cell
Lung monocyte
Marrow late pro−B cell
Marrow B cell
Thymus immature T cell
Marrow precursor B cell
Brain Non−Myeloid neuron
Pancreas pancreatic A cell
Pancreas pancreatic PP cell
Bladder bladder cell
Pancreas pancreatic D cell
Mammary Gland stromal cell
Heart cardiac muscle cell
Trachea mesenchymal cell
Brain Non−Myeloid brain pericyte
Pancreas pancreatic stellate cell
Limb Muscle mesenchymal stem cell
Fat mesenchymal stem cell of adipose
Brain Non−Myeloid endothelial cell
Pancreas pancreatic ductal cell
Lung unknown cell type
Large Intestine enteroendocrine cell
Lung stromal cell
Trachea epithelial cell
Heart smooth muscle cell
Heart fibroblast
Fat unknown cell type
Brain Non−Myeloid astrocyte
Mammary Gland endothelial cell
Liver endothelial cell of hepatic sinusoid
Lung ciliated columnar cell of tracheobronchial tree
Tongue keratinocyte
Brain Non−Myeloid Bergmann glial cell
Fat endothelial cell
Mammary Gland basal cell
Trachea endothelial cell
Heart endocardial cell
Pancreas pancreatic acinar cell
Limb Muscle skeletal muscle satellite cell
Heart unknown cell type
Liver hepatocyte
Trachea blood cell
Large Intestine enterocyte of epithelium of large intestine
Bladder bladder urothelial cell
Heart myofibroblast cell
Lung epithelial cell of lung
Tongue basal cell of epidermis
Limb Muscle endothelial cell
Skin keratinocyte stem cell
Pancreas endothelial cell
Lung lung endothelial cell
Skin epidermal cell
Mammary Gland luminal epithelial cell of mammary gland
Heart erythrocyte
Skin basal cell of epidermis
Kidney kidney collecting duct epithelial cell
Kidney epithelial cell of proximal tubule
Skin stem cell of epidermis
Heart endothelial cell
Large Intestine epithelial cell of large intestine
Kidney endothelial cell
Large Intestine Brush cell of epithelium proper of large intestine

**Supplementary Fig. 1: CELLECT-MAGMA cellular associations with IEAA (immune cells and precursors, non-immune cells)**

Results from CELLECT-MAGMA cell-type enrichment analysis of IEAA. Cell types are split into immune cells and immune cell precursors (top half of figure) and non-immune cells (bottom half of figure). Circles represent negative, log-transformed, unadjusted P values. Statistical analyses were conducted using one-sided t-tests. Blue circles represent cell types significantly enriched in IEAA-associated SNPs at a FDR of 0.05. Black circles represent non-significant associations. Black circles to the right of the dotted line represent nominally significant associations (i.e., cells with P value < 0.05 that fail to survive correction for multiple comparisons). Exact P values are contained in Supplementary Data 30.

CELLECT: CELL-type Expression-specific integration for Complex Traits; MAGMA: Multi-marker Analysis of GenoMic Annotation; IEAA: intrinsic epigenetic age acceleration; SNP: single nucleotide polymorphism; FDR: false discovery rate.

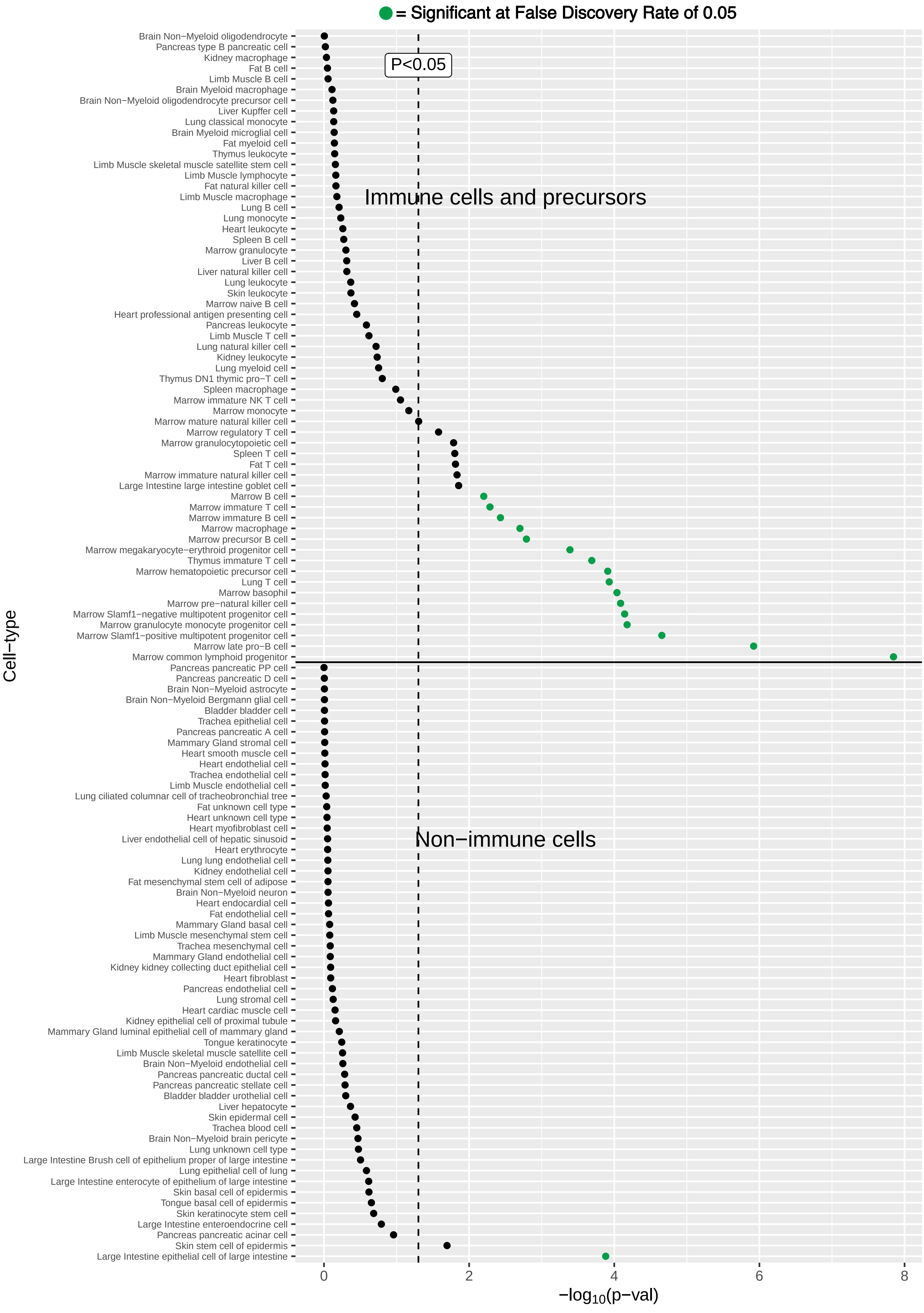
= Significant at False Discovery Rate of 0.05
P<0.05
Immune cells and precursors
Non−immune cells
Cell−type
−log10(p−val)
0
2
4
6
8
Brain Non−Myeloid oligodendrocyte
Pancreas type B pancreatic cell
Kidney macrophage
Fat B cell
Limb Muscle B cell
Brain Myeloid macrophage
Brain Non−Myeloid oligodendrocyte precursor cell
Liver Kupffer cell
Lung classical monocyte
Brain Myeloid microglial cell
Fat myeloid cell
Thymus leukocyte
Limb Muscle skeletal muscle satellite stem cell
Limb Muscle lymphocyte
Fat natural killer cell
Limb Muscle macrophage
Lung B cell
Lung monocyte
Heart leukocyte
Spleen B cell
Marrow granulocyte
Liver B cell
Liver natural killer cell
Lung leukocyte
Skin leukocyte
Marrow naive B cell
Heart professional antigen presenting cell
Pancreas leukocyte
Limb Muscle T cell
Lung natural killer cell
Kidney leukocyte
Lung myeloid cell
Thymus DN1 thymic pro−T cell
Spleen macrophage
Marrow immature NK T cell
Marrow monocyte
Marrow mature natural killer cell
Marrow regulatory T cell
Marrow granulocytopoietic cell
Spleen T cell
Fat T cell
Marrow immature natural killer cell
Large Intestine large intestine goblet cell
Marrow B cell
Marrow immature T cell
Marrow immature B cell
Marrow macrophage
Marrow precursor B cell
Marrow megakaryocyte−erythroid progenitor cell
Thymus immature T cell
Marrow hematopoietic precursor cell
Lung T cell
Marrow basophil
Marrow pre−natural killer cell
Marrow Slamf1−negative multipotent progenitor cell
Marrow granulocyte monocyte progenitor cell
Marrow Slamf1−positive multipotent progenitor cell
Marrow late pro−B cell
Marrow common lymphoid progenitor
Pancreas pancreatic PP cell
Pancreas pancreatic D cell
Brain Non−Myeloid astrocyte
Brain Non−Myeloid Bergmann glial cell
Bladder bladder cell
Trachea epithelial cell
Pancreas pancreatic A cell
Mammary Gland stromal cell
Heart smooth muscle cell
Heart endothelial cell
Trachea endothelial cell
Limb Muscle endothelial cell
Lung ciliated columnar cell of tracheobronchial tree
Fat unknown cell type
Heart unknown cell type
Heart myofibroblast cell
Liver endothelial cell of hepatic sinusoid
Heart erythrocyte
Lung lung endothelial cell
Kidney endothelial cell
Fat mesenchymal stem cell of adipose
Brain Non−Myeloid neuron
Heart endocardial cell
Fat endothelial cell
Mammary Gland basal cell
Limb Muscle mesenchymal stem cell
Trachea mesenchymal cell
Mammary Gland endothelial cell
Kidney kidney collecting duct epithelial cell
Heart fibroblast
Pancreas endothelial cell
Lung stromal cell
Heart cardiac muscle cell
Kidney epithelial cell of proximal tubule
Mammary Gland luminal epithelial cell of mammary gland
Tongue keratinocyte
Limb Muscle skeletal muscle satellite cell
Brain Non−Myeloid endothelial cell
Pancreas pancreatic ductal cell
Pancreas pancreatic stellate cell
Bladder bladder urothelial cell
Liver hepatocyte
Skin epidermal cell
Trachea blood cell
Brain Non−Myeloid brain pericyte
Lung unknown cell type
Large Intestine Brush cell of epithelium proper of large intestine
Lung epithelial cell of lung
Large Intestine enterocyte of epithelium of large intestine
Skin basal cell of epidermis
Tongue basal cell of epidermis
Skin keratinocyte stem cell
Large Intestine enteroendocrine cell
Pancreas pancreatic acinar cell
Skin stem cell of epidermis
Large Intestine epithelial cell of large intestine

**Supplementary Fig. 2: CELLECT-MAGMA cellular associations with HannumAge (immune cells and precursors, non-immune cells)**

Results from CELLECT-MAGMA cell-type enrichment analysis of HannumAge. Cell types are split into immune cells and immune cell precursors (top half of figure) and non-immune cells (bottom half of figure). Circles represent negative, log-transformed, unadjusted P values. Statistical analyses were conducted using one-sided t-tests. Green circles represent cell types significantly enriched in HannumAge-associated SNPs at a FDR of 0.05. Black circles represent non-significant associations. Black circles to the right of the dotted line represent nominally significant associations (i.e., cells with P value < 0.05 that fail to survive correction for multiple comparisons). Exact P values are contained in Supplementary Data 31.

CELLECT: CELL-type Expression-specific integration for Complet Traits; MAGMA: Multi-marker Analysis of GenoMic Annotation; SNP: single nucleotide polymorphism; FDR: false discovery rate.

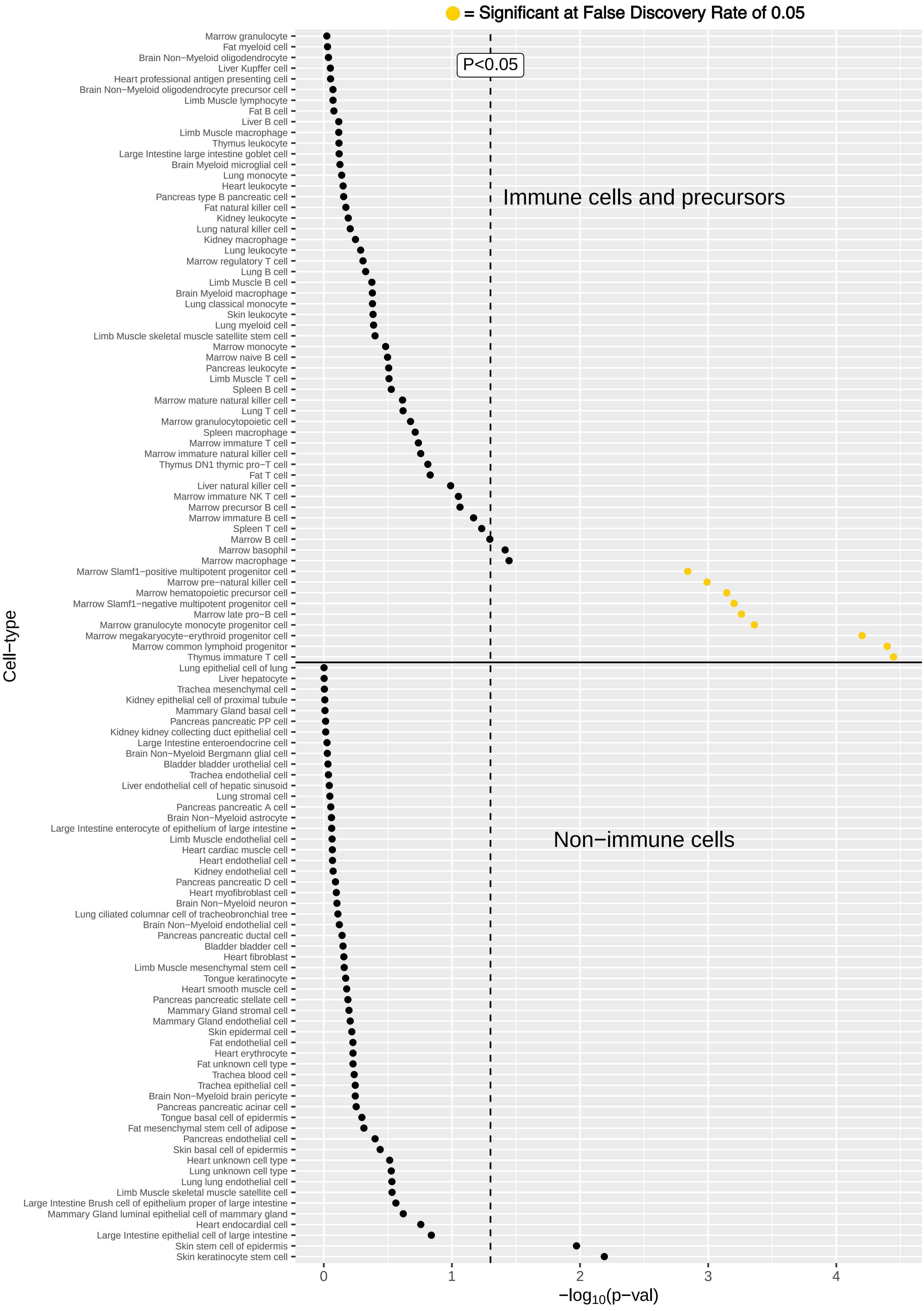
= Significant at False Discovery Rate of 0.05
P<0.05
Immune cells and precursors
Non−immune cells
Cell−type
−log10(p−val)
0
1
2
3
4
Marrow granulocyte
Fat myeloid cell
Brain Non−Myeloid oligodendrocyte
Liver Kupffer cell
Heart professional antigen presenting cell
Brain Non−Myeloid oligodendrocyte precursor cell
Limb Muscle lymphocyte
Fat B cell
Liver B cell
Limb Muscle macrophage
Thymus leukocyte
Large Intestine large intestine goblet cell
Brain Myeloid microglial cell
Lung monocyte
Heart leukocyte
Pancreas type B pancreatic cell
Fat natural killer cell
Kidney leukocyte
Lung natural killer cell
Kidney macrophage
Lung leukocyte
Marrow regulatory T cell
Lung B cell
Limb Muscle B cell
Brain Myeloid macrophage
Lung classical monocyte
Skin leukocyte
Lung myeloid cell
Limb Muscle skeletal muscle satellite stem cell
Marrow monocyte
Marrow naive B cell
Pancreas leukocyte
Limb Muscle T cell
Spleen B cell
Marrow mature natural killer cell
Lung T cell
Marrow granulocytopoietic cell
Spleen macrophage
Marrow immature T cell
Marrow immature natural killer cell
Thymus DN1 thymic pro−T cell
Fat T cell
Liver natural killer cell
Marrow immature NK T cell
Marrow precursor B cell
Marrow immature B cell
Spleen T cell
Marrow B cell
Marrow basophil
Marrow macrophage
Marrow Slamf1−positive multipotent progenitor cell
Marrow pre−natural killer cell
Marrow hematopoietic precursor cell
Marrow Slamf1−negative multipotent progenitor cell
Marrow late pro−B cell
Marrow granulocyte monocyte progenitor cell
Marrow megakaryocyte−erythroid progenitor cell
Marrow common lymphoid progenitor
Thymus immature T cell
Lung epithelial cell of lung
Liver hepatocyte
Trachea mesenchymal cell
Kidney epithelial cell of proximal tubule
Mammary Gland basal cell
Pancreas pancreatic PP cell
Kidney kidney collecting duct epithelial cell
Large Intestine enteroendocrine cell
Brain Non−Myeloid Bergmann glial cell
Bladder bladder urothelial cell
Trachea endothelial cell
Liver endothelial cell of hepatic sinusoid
Lung stromal cell
Pancreas pancreatic A cell
Brain Non−Myeloid astrocyte
Large Intestine enterocyte of epithelium of large intestine
Limb Muscle endothelial cell
Heart cardiac muscle cell
Heart endothelial cell
Kidney endothelial cell
Pancreas pancreatic D cell
Heart myofibroblast cell
Brain Non−Myeloid neuron
Lung ciliated columnar cell of tracheobronchial tree
Brain Non−Myeloid endothelial cell
Pancreas pancreatic ductal cell
Bladder bladder cell
Heart fibroblast
Limb Muscle mesenchymal stem cell
Tongue keratinocyte
Heart smooth muscle cell
Pancreas pancreatic stellate cell
Mammary Gland stromal cell
Mammary Gland endothelial cell
Skin epidermal cell
Fat endothelial cell
Heart erythrocyte
Fat unknown cell type
Trachea blood cell
Trachea epithelial cell
Brain Non−Myeloid brain pericyte
Pancreas pancreatic acinar cell
Tongue basal cell of epidermis
Fat mesenchymal stem cell of adipose
Pancreas endothelial cell
Skin basal cell of epidermis
Heart unknown cell type
Lung unknown cell type
Lung lung endothelial cell
Limb Muscle skeletal muscle satellite cell
Large Intestine Brush cell of epithelium proper of large intestine
Mammary Gland luminal epithelial cell of mammary gland
Heart endocardial cell
Large Intestine epithelial cell of large intestine
Skin stem cell of epidermis
Skin keratinocyte stem cell

**Supplementary Fig. 3: CELLECT-MAGMA cellular associations with GrimAge (immune cells and precursors, non-immune cells)**

Results from CELLECT-MAGMA cell-type enrichment analysis of GrimAge. Cell types are split into immune cells and immune cell precursors (top half of figure) and non-immune cells (bottom half of figure). Circles represent negative, log-transformed, unadjusted P values. Statistical analyses were conducted using one-sided t-tests. Yellow circles represent cell types significantly enriched in GrimAge-associated SNPs at a FDR of 0.05. Black circles represent non-significant associations. Black circles to the right of the dotted line represent nominally significant associations (i.e., cells with P value < 0.05 that fail to survive correction for multiple comparisons). Exact P values are contained in Supplementary Data 32.

CELLECT: CELL-type Expression-specific integration for Complex Traits; MAGMA: Multi-marker Analysis of GenoMic Annotation; SNP: single nucleotide polymorphism; FDR: false discovery rate.

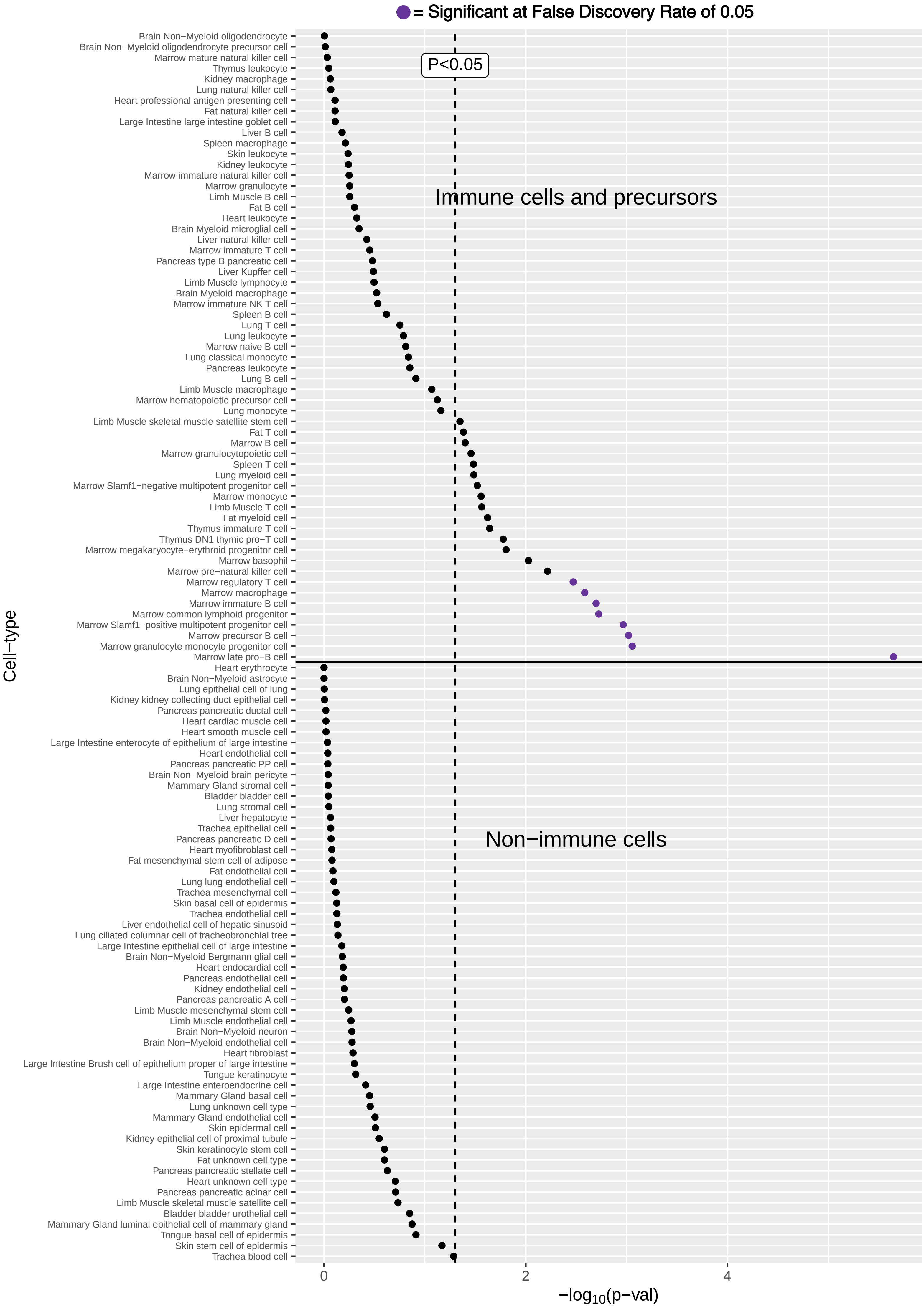
= Significant at False Discovery Rate of 0.05
P<0.05
Immune cells and precursors
Non−immune cells
Cell−type
−log10(p−val)
0
2
4
Brain Non−Myeloid oligodendrocyte
Brain Non−Myeloid oligodendrocyte precursor cell
Marrow mature natural killer cell
Thymus leukocyte
Kidney macrophage
Lung natural killer cell
Heart professional antigen presenting cell
Fat natural killer cell
Large Intestine large intestine goblet cell
Liver B cell
Spleen macrophage
Skin leukocyte
Kidney leukocyte
Marrow immature natural killer cell
Marrow granulocyte
Limb Muscle B cell
Fat B cell
Heart leukocyte
Brain Myeloid microglial cell
Liver natural killer cell
Marrow immature T cell
Pancreas type B pancreatic cell
Liver Kupffer cell
Limb Muscle lymphocyte
Brain Myeloid macrophage
Marrow immature NK T cell
Spleen B cell
Lung T cell
Lung leukocyte
Marrow naive B cell
Lung classical monocyte
Pancreas leukocyte
Lung B cell
Limb Muscle macrophage
Marrow hematopoietic precursor cell
Lung monocyte
Limb Muscle skeletal muscle satellite stem cell
Fat T cell
Marrow B cell
Marrow granulocytopoietic cell
Spleen T cell
Lung myeloid cell
Marrow Slamf1−negative multipotent progenitor cell
Marrow monocyte
Limb Muscle T cell
Fat myeloid cell
Thymus immature T cell
Thymus DN1 thymic pro−T cell
Marrow megakaryocyte−erythroid progenitor cell
Marrow basophil
Marrow pre−natural killer cell
Marrow regulatory T cell
Marrow macrophage
Marrow immature B cell
Marrow common lymphoid progenitor
Marrow Slamf1−positive multipotent progenitor cell
Marrow precursor B cell
Marrow granulocyte monocyte progenitor cell
Marrow late pro−B cell
Heart erythrocyte
Brain Non−Myeloid astrocyte
Lung epithelial cell of lung
Kidney kidney collecting duct epithelial cell
Pancreas pancreatic ductal cell
Heart cardiac muscle cell
Heart smooth muscle cell
Large Intestine enterocyte of epithelium of large intestine
Heart endothelial cell
Pancreas pancreatic PP cell
Brain Non−Myeloid brain pericyte
Mammary Gland stromal cell
Bladder bladder cell
Lung stromal cell
Liver hepatocyte
Trachea epithelial cell
Pancreas pancreatic D cell
Heart myofibroblast cell
Fat mesenchymal stem cell of adipose
Fat endothelial cell
Lung lung endothelial cell
Trachea mesenchymal cell
Skin basal cell of epidermis
Trachea endothelial cell
Liver endothelial cell of hepatic sinusoid
Lung ciliated columnar cell of tracheobronchial tree
Large Intestine epithelial cell of large intestine
Brain Non−Myeloid Bergmann glial cell
Heart endocardial cell
Pancreas endothelial cell
Kidney endothelial cell
Pancreas pancreatic A cell
Limb Muscle mesenchymal stem cell
Limb Muscle endothelial cell
Brain Non−Myeloid neuron
Brain Non−Myeloid endothelial cell
Heart fibroblast
Large Intestine Brush cell of epithelium proper of large intestine
Tongue keratinocyte
Large Intestine enteroendocrine cell
Mammary Gland basal cell
Lung unknown cell type
Mammary Gland endothelial cell
Skin epidermal cell
Kidney epithelial cell of proximal tubule
Skin keratinocyte stem cell
Fat unknown cell type
Pancreas pancreatic stellate cell
Heart unknown cell type
Pancreas pancreatic acinar cell
Limb Muscle skeletal muscle satellite cell
Bladder bladder urothelial cell
Mammary Gland luminal epithelial cell of mammary gland
Tongue basal cell of epidermis
Skin stem cell of epidermis
Trachea blood cell

**Supplementary Fig. 4: CELLECT-MAGMA cellular associations with PhenoAge (immune cells and precursors, non-immune cells)**

Results from CELLECT-MAGMA cell-type enrichment analysis of PhenoAge. Cell types are split into immune cells and immune cell precursors (top half of figure) and non-immune cells (bottom half of figure). Circles represent negative, log-transformed, unadjusted P values. Statistical analyses were conducted using one-sided t-tests. Purple circles represent cell types significantly enriched in PhenoAge-associated SNPs at a FDR of 0.05. Black circles represent non-significant associations. Black circles to the right of the dotted line represent nominally significant associations (i.e., cells with P value < 0.05 that fail to survive correction for multiple comparisons). Exact P values are contained in Supplementary Data 33.

CELLECT: CELL-type Expression-specific integration for Complex Traits; MAGMA: Multi-marker Analysis of GenoMic Annotation; SNP: single nucleotide polymorphism; FDR: false discovery rate.

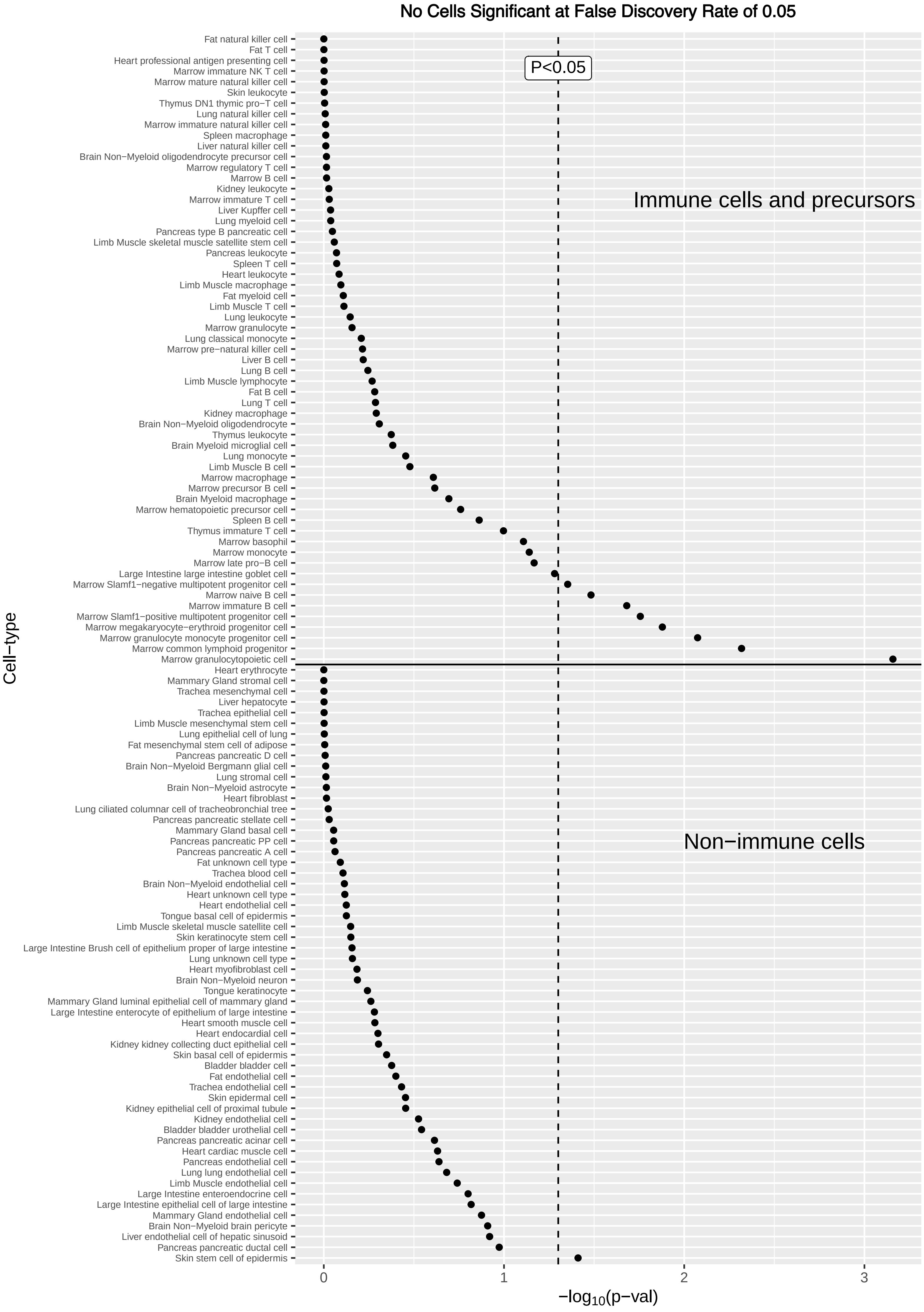
No Cells Significant at False Discovery Rate of 0.05
P<0.05
Immune cells and precursors
Non−immune cells
Cell−type
−log10(p−val)
Fat natural killer cell
Fat T cell
Heart professional antigen presenting cell
Marrow immature NK T cell
Marrow mature natural killer cell
Skin leukocyte
Thymus DN1 thymic pro−T cell
Lung natural killer cell
Marrow immature natural killer cell
Spleen macrophage
Liver natural killer cell
Brain Non−Myeloid oligodendrocyte precursor cell
Marrow regulatory T cell
Marrow B cell
Kidney leukocyte
Marrow immature T cell
Liver Kupffer cell
Lung myeloid cell
Pancreas type B pancreatic cell
Limb Muscle skeletal muscle satellite stem cell
Pancreas leukocyte
Spleen T cell
Heart leukocyte
Limb Muscle macrophage
Fat myeloid cell
Limb Muscle T cell
Lung leukocyte
Marrow granulocyte
Lung classical monocyte
Marrow pre−natural killer cell
Liver B cell
Lung B cell
Limb Muscle lymphocyte
Fat B cell
Lung T cell
Kidney macrophage
Brain Non−Myeloid oligodendrocyte
Thymus leukocyte
Brain Myeloid microglial cell
Lung monocyte
Limb Muscle B cell
Marrow macrophage
Marrow precursor B cell
Brain Myeloid macrophage
Marrow hematopoietic precursor cell
Spleen B cell
Thymus immature T cell
Marrow basophil
Marrow monocyte
Marrow late pro−B cell
Large Intestine large intestine goblet cell
Marrow Slamf1−negative multipotent progenitor cell
Marrow naive B cell
Marrow immature B cell
Marrow Slamf1−positive multipotent progenitor cell
Marrow megakaryocyte−erythroid progenitor cell
Marrow granulocyte monocyte progenitor cell
Marrow common lymphoid progenitor
Marrow granulocytopoietic cell
Heart erythrocyte
Mammary Gland stromal cell
Trachea mesenchymal cell
Liver hepatocyte
Trachea epithelial cell
Limb Muscle mesenchymal stem cell
Lung epithelial cell of lung
Fat mesenchymal stem cell of adipose
Pancreas pancreatic D cell
Brain Non−Myeloid Bergmann glial cell
Lung stromal cell
Brain Non−Myeloid astrocyte
Heart fibroblast
Lung ciliated columnar cell of tracheobronchial tree
Pancreas pancreatic stellate cell
Mammary Gland basal cell
Pancreas pancreatic PP cell
Pancreas pancreatic A cell
Fat unknown cell type
Trachea blood cell
Brain Non−Myeloid endothelial cell
Heart unknown cell type
Heart endothelial cell
Tongue basal cell of epidermis
Limb Muscle skeletal muscle satellite cell
Skin keratinocyte stem cell
Large Intestine Brush cell of epithelium proper of large intestine
Lung unknown cell type
Heart myofibroblast cell
Brain Non−Myeloid neuron
Tongue keratinocyte
Mammary Gland luminal epithelial cell of mammary gland
Large Intestine enterocyte of epithelium of large intestine
Heart smooth muscle cell
Heart endocardial cell
Kidney kidney collecting duct epithelial cell
Skin basal cell of epidermis
Bladder bladder cell
Fat endothelial cell
Trachea endothelial cell
Skin epidermal cell
Kidney epithelial cell of proximal tubule
Kidney endothelial cell
Bladder bladder urothelial cell
Pancreas pancreatic acinar cell
Heart cardiac muscle cell
Pancreas endothelial cell
Lung lung endothelial cell
Limb Muscle endothelial cell
Large Intestine enteroendocrine cell
Large Intestine epithelial cell of large intestine
Mammary Gland endothelial cell
Brain Non−Myeloid brain pericyte
Liver endothelial cell of hepatic sinusoid
Pancreas pancreatic ductal cell
Skin stem cell of epidermis
0
1
2
3

**Supplementary Fig. 5: CELLECT-MAGMA cellular associations with multivariate longevity (immune cells and precursors, non-immune cells)**

Results from CELLECT-MAGMA cell-type enrichment analysis of multivariate longevity. Cell types are split into immune cells and immune cell precursors (top half of figure) and non-immune cells (bottom half of figure). Circles represent negative, log-transformed, unadjusted P values. Statistical analyses were conducted using one-sided t-tests. Black circles represent non-significant cell-trait associations at a FDR of 0.05. Black circles to the right of the dotted line represent nominally significant associations (i.e., cells with P value < 0.05 that fail to survive correction for multiple comparisons). Exact P values are contained in Supplementary Data 34.

CELLECT: CELL-type Expression-specific integration for Complex Traits; MAGMA: Multi-marker Analysis of GenoMic Annotation; SNP: single nucleotide polymorphism; FDR: false discovery rate.

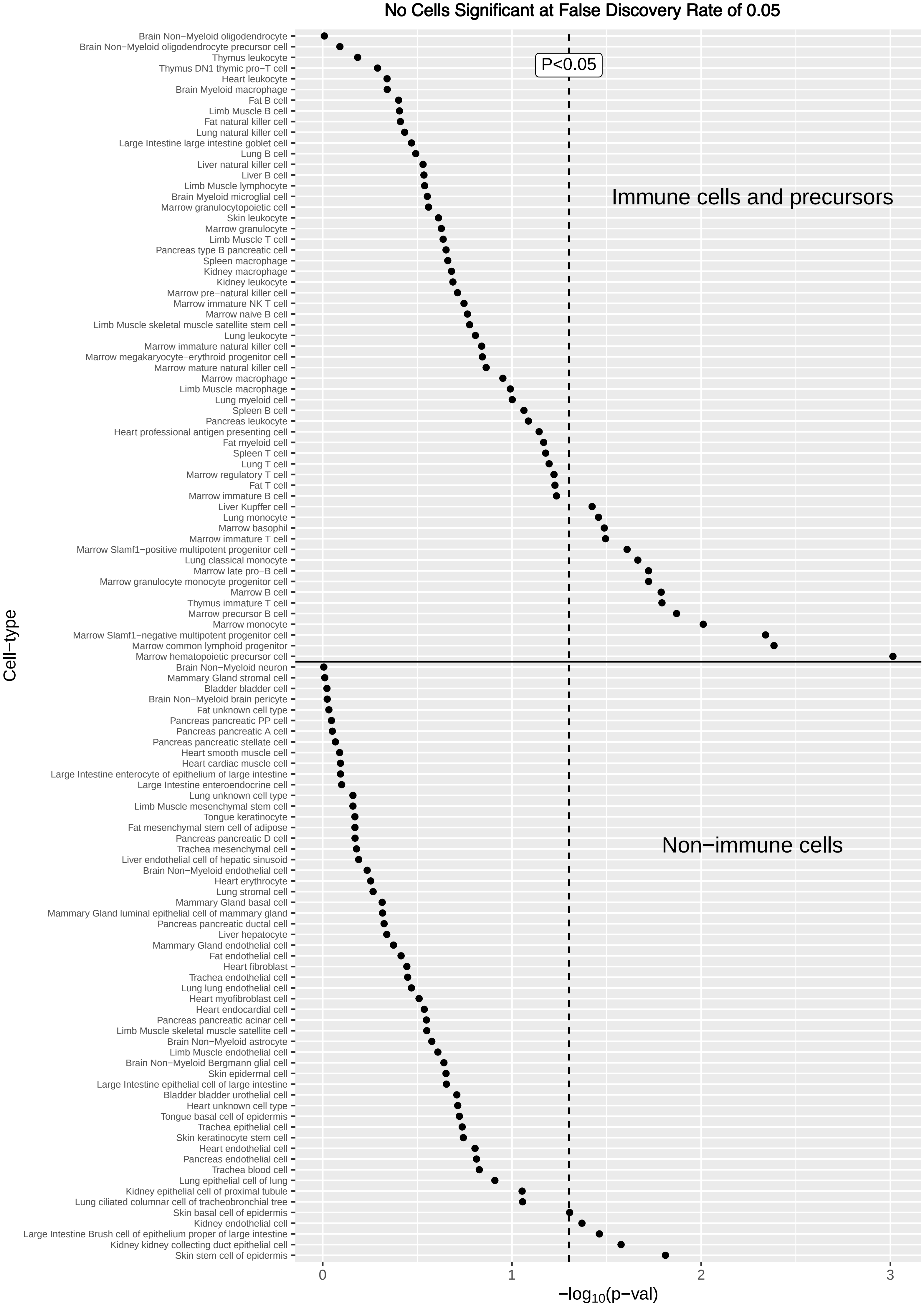
No Cells Significant at False Discovery Rate of 0.05
P<0.05
Immune cells and precursors
Non−immune cells
Cell−type
−log10(p−val)
0
1
2
3
Brain Non−Myeloid oligodendrocyte
Brain Non−Myeloid oligodendrocyte precursor cell
Thymus leukocyte
Thymus DN1 thymic pro−T cell
Heart leukocyte
Brain Myeloid macrophage
Fat B cell
Limb Muscle B cell
Fat natural killer cell
Lung natural killer cell
Large Intestine large intestine goblet cell
Lung B cell
Liver natural killer cell
Liver B cell
Limb Muscle lymphocyte
Brain Myeloid microglial cell
Marrow granulocytopoietic cell
Skin leukocyte
Marrow granulocyte
Limb Muscle T cell
Pancreas type B pancreatic cell
Spleen macrophage
Kidney macrophage
Kidney leukocyte
Marrow pre−natural killer cell
Marrow immature NK T cell
Marrow naive B cell
Limb Muscle skeletal muscle satellite stem cell
Lung leukocyte
Marrow immature natural killer cell
Marrow megakaryocyte−erythroid progenitor cell
Marrow mature natural killer cell
Marrow macrophage
Limb Muscle macrophage
Lung myeloid cell
Spleen B cell
Pancreas leukocyte
Heart professional antigen presenting cell
Fat myeloid cell
Spleen T cell
Lung T cell
Marrow regulatory T cell
Fat T cell
Marrow immature B cell
Liver Kupffer cell
Lung monocyte
Marrow basophil
Marrow immature T cell
Marrow Slamf1−positive multipotent progenitor cell
Lung classical monocyte
Marrow late pro−B cell
Marrow granulocyte monocyte progenitor cell
Marrow B cell
Thymus immature T cell
Marrow precursor B cell
Marrow monocyte
Marrow Slamf1−negative multipotent progenitor cell
Marrow common lymphoid progenitor
Marrow hematopoietic precursor cell
Brain Non−Myeloid neuron
Mammary Gland stromal cell
Bladder bladder cell
Brain Non−Myeloid brain pericyte
Fat unknown cell type
Pancreas pancreatic PP cell
Pancreas pancreatic A cell
Pancreas pancreatic stellate cell
Heart smooth muscle cell
Heart cardiac muscle cell
Large Intestine enterocyte of epithelium of large intestine
Large Intestine enteroendocrine cell
Lung unknown cell type
Limb Muscle mesenchymal stem cell
Tongue keratinocyte
Fat mesenchymal stem cell of adipose
Pancreas pancreatic D cell
Trachea mesenchymal cell
Liver endothelial cell of hepatic sinusoid
Brain Non−Myeloid endothelial cell
Heart erythrocyte
Lung stromal cell
Mammary Gland basal cell
Mammary Gland luminal epithelial cell of mammary gland
Pancreas pancreatic ductal cell
Liver hepatocyte
Mammary Gland endothelial cell
Fat endothelial cell
Heart fibroblast
Trachea endothelial cell
Lung lung endothelial cell
Heart myofibroblast cell
Heart endocardial cell
Pancreas pancreatic acinar cell
Limb Muscle skeletal muscle satellite cell
Brain Non−Myeloid astrocyte
Limb Muscle endothelial cell
Brain Non−Myeloid Bergmann glial cell
Skin epidermal cell
Large Intestine epithelial cell of large intestine
Bladder bladder urothelial cell
Heart unknown cell type
Tongue basal cell of epidermis
Trachea epithelial cell
Skin keratinocyte stem cell
Heart endothelial cell
Pancreas endothelial cell
Trachea blood cell
Lung epithelial cell of lung
Kidney epithelial cell of proximal tubule
Lung ciliated columnar cell of tracheobronchial tree
Skin basal cell of epidermis
Kidney endothelial cell
Large Intestine Brush cell of epithelium proper of large intestine
Kidney kidney collecting duct epithelial cell
Skin stem cell of epidermis

**Supplementary Fig. 6: CELLECT-LDSC cellular associations with IEAA (immune cells and precursors, non-immune cells)**

Results from CELLECT-LDSC cell-type enrichment analysis of multivariate longevity. Cell types are split into immune cells and immune cell precursors (top half of figure) and non-immune cells (bottom half of figure). Circles represent negative, log-transformed, unadjusted P values. Statistical analyses were conducted using one-sided t-tests. Black circles represent non-significant cell-trait associations at a FDR of 0.05. Black circles to the right of the dotted line represent nominally significant associations (i.e., cells with P value < 0.05 that fail to survive correction for multiple comparisons). Exact P values are contained in Supplementary Data 30.

CELLECT: CELL-type Expression-specific integration for Complex Traits; LDSC: LD Score Regression; IEAA: intrinsic epigenetic age acceleration; SNP: single nucleotide polymorphism; FDR: false discovery rate.

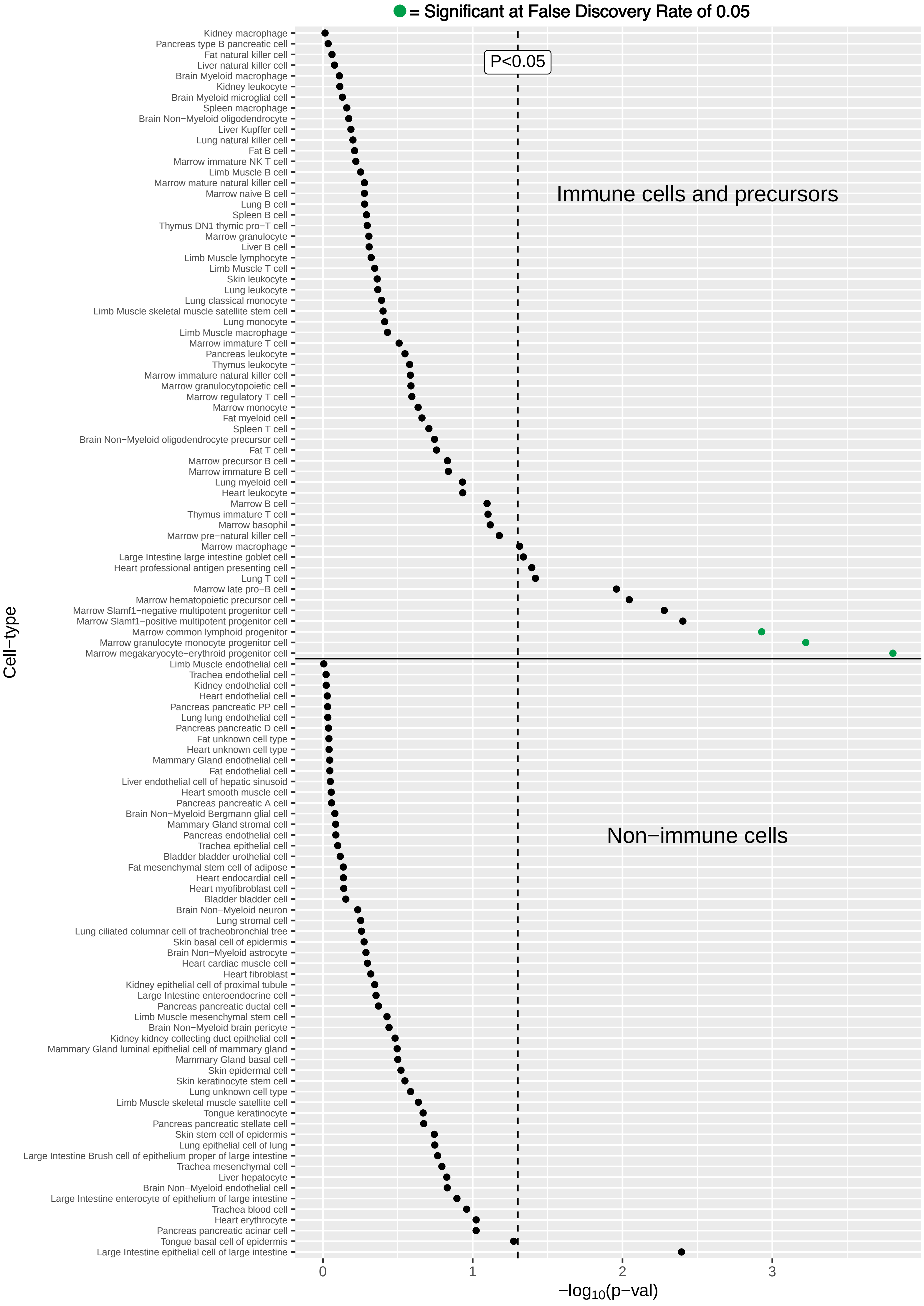

= Significant at False Discovery Rate of 0.05
P<0.05
Immune cells and precursors
Non−immune cells
Cell−type
−log10(p−val)
0
1
2
3
Kidney macrophage
Pancreas type B pancreatic cell
Fat natural killer cell
Liver natural killer cell
Brain Myeloid macrophage
Kidney leukocyte
Brain Myeloid microglial cell
Spleen macrophage
Brain Non−Myeloid oligodendrocyte
Liver Kupffer cell
Lung natural killer cell
Fat B cell
Marrow immature NK T cell
Limb Muscle B cell
Marrow mature natural killer cell
Marrow naive B cell
Lung B cell
Spleen B cell
Thymus DN1 thymic pro−T cell
Marrow granulocyte
Liver B cell
Limb Muscle lymphocyte
Limb Muscle T cell
Skin leukocyte
Lung leukocyte
Lung classical monocyte
Limb Muscle skeletal muscle satellite stem cell
Lung monocyte
Limb Muscle macrophage
Marrow immature T cell
Pancreas leukocyte
Thymus leukocyte
Marrow immature natural killer cell
Marrow granulocytopoietic cell
Marrow regulatory T cell
Marrow monocyte
Fat myeloid cell
Spleen T cell
Brain Non−Myeloid oligodendrocyte precursor cell
Fat T cell
Marrow precursor B cell
Marrow immature B cell
Lung myeloid cell
Heart leukocyte
Marrow B cell
Thymus immature T cell
Marrow basophil
Marrow pre−natural killer cell
Marrow macrophage
Large Intestine large intestine goblet cell
Heart professional antigen presenting cell
Lung T cell
Marrow late pro−B cell
Marrow hematopoietic precursor cell
Marrow Slamf1−negative multipotent progenitor cell
Marrow Slamf1−positive multipotent progenitor cell
Marrow common lymphoid progenitor
Marrow granulocyte monocyte progenitor cell
Marrow megakaryocyte−erythroid progenitor cell
Limb Muscle endothelial cell
Trachea endothelial cell
Kidney endothelial cell
Heart endothelial cell
Pancreas pancreatic PP cell
Lung lung endothelial cell
Pancreas pancreatic D cell
Fat unknown cell type
Heart unknown cell type
Mammary Gland endothelial cell
Fat endothelial cell
Liver endothelial cell of hepatic sinusoid
Heart smooth muscle cell
Pancreas pancreatic A cell
Brain Non−Myeloid Bergmann glial cell
Mammary Gland stromal cell
Pancreas endothelial cell
Trachea epithelial cell
Bladder bladder urothelial cell
Fat mesenchymal stem cell of adipose
Heart endocardial cell
Heart myofibroblast cell
Bladder bladder cell
Brain Non−Myeloid neuron
Lung stromal cell
Lung ciliated columnar cell of tracheobronchial tree
Skin basal cell of epidermis
Brain Non−Myeloid astrocyte
Heart cardiac muscle cell
Heart fibroblast
Kidney epithelial cell of proximal tubule
Large Intestine enteroendocrine cell
Pancreas pancreatic ductal cell
Limb Muscle mesenchymal stem cell
Brain Non−Myeloid brain pericyte
Kidney kidney collecting duct epithelial cell
Mammary Gland luminal epithelial cell of mammary gland
Mammary Gland basal cell
Skin epidermal cell
Skin keratinocyte stem cell
Lung unknown cell type
Limb Muscle skeletal muscle satellite cell
Tongue keratinocyte
Pancreas pancreatic stellate cell
Skin stem cell of epidermis
Lung epithelial cell of lung
Large Intestine Brush cell of epithelium proper of large intestine
Trachea mesenchymal cell
Liver hepatocyte
Brain Non−Myeloid endothelial cell
Large Intestine enterocyte of epithelium of large intestine
Trachea blood cell
Heart erythrocyte
Pancreas pancreatic acinar cell
Tongue basal cell of epidermis
Large Intestine epithelial cell of large intestine

**Supplementary Fig. 7: CELLECT-LDSC cellular associations with HannumAge (immune cells and precursors, non-immune cells)**

Results from CELLECT-LDSC cell-type enrichment analysis of HannumAge. Cell types are split into immune cells and immune cell precursors (top half of figure) and non-immune cells (bottom half of figure). Circles represent negative, log-transformed, unadjusted P values. Statistical analyses were conducted using one-sided t-tests. Green circles represent cell types significantly enriched in HannumAge-associated SNPs at a FDR of 0.05. Black circles represent non-significant associations. Black circles to the right of the dotted line represent nominally significant associations (i.e., cells with P value $< 0.05$ that fail to survive correction for multiple comparisons). Exact P values are contained in Supplementary Data 31.

CELLECT: CELL-type Expression-specific integration for Complex Traits; LDSC: LD Score Regression; SNP: single nucleotide polymorphism; FDR: false discovery rate.

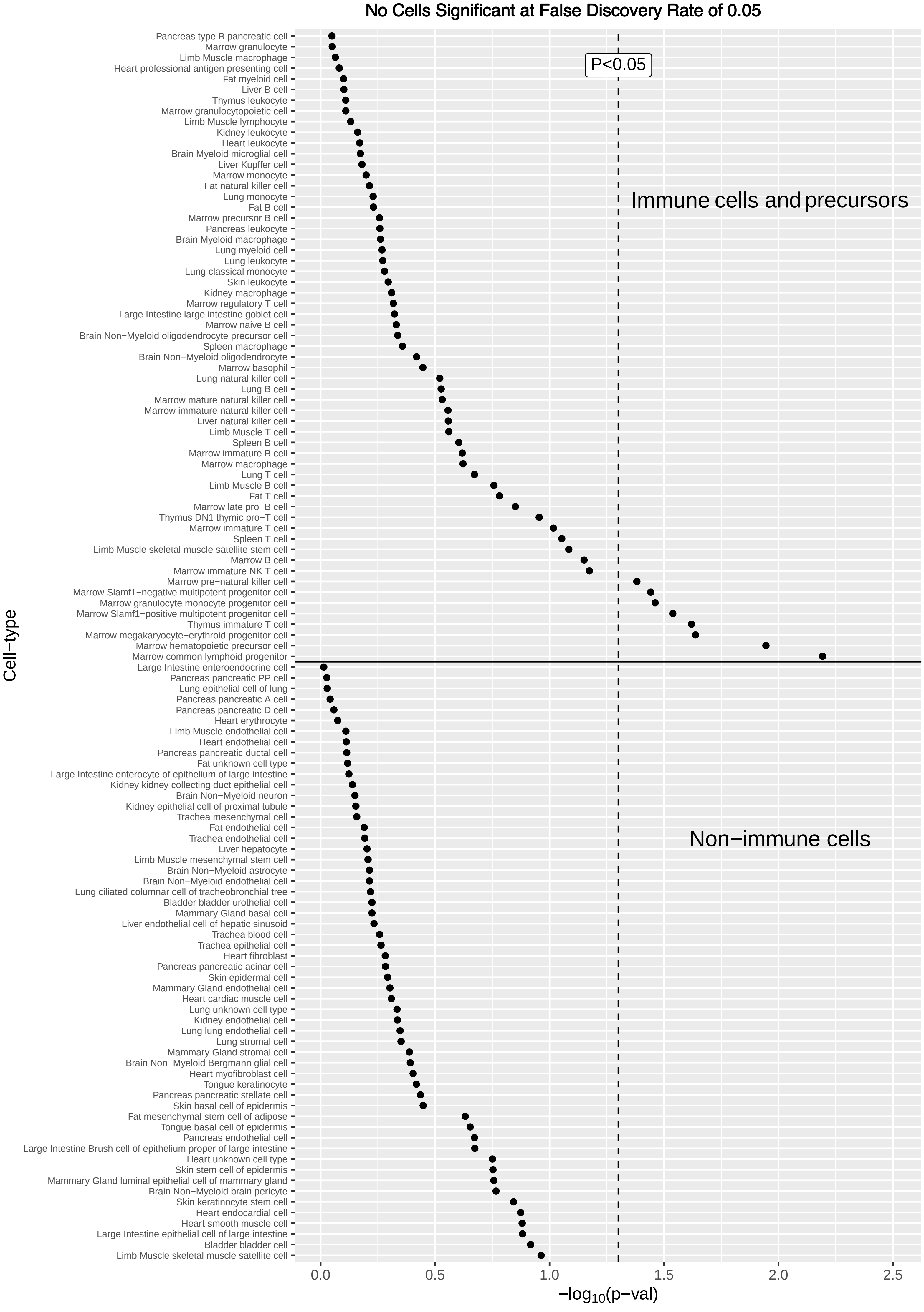
No Cells Significant at False Discovery Rate of 0.05
P<0.05
Immune cells and precursors
Non−immune cells
Cell−type
−log10(p−val)
0.0
0.5
1.0
1.5
2.0
2.5
Pancreas type B pancreatic cell
Marrow granulocyte
Limb Muscle macrophage
Heart professional antigen presenting cell
Fat myeloid cell
Liver B cell
Thymus leukocyte
Marrow granulocytopoietic cell
Limb Muscle lymphocyte
Kidney leukocyte
Heart leukocyte
Brain Myeloid microglial cell
Liver Kupffer cell
Marrow monocyte
Fat natural killer cell
Lung monocyte
Fat B cell
Marrow precursor B cell
Pancreas leukocyte
Brain Myeloid macrophage
Lung myeloid cell
Lung leukocyte
Lung classical monocyte
Skin leukocyte
Kidney macrophage
Marrow regulatory T cell
Large Intestine large intestine goblet cell
Marrow naive B cell
Brain Non−Myeloid oligodendrocyte precursor cell
Spleen macrophage
Brain Non−Myeloid oligodendrocyte
Marrow basophil
Lung natural killer cell
Lung B cell
Marrow mature natural killer cell
Marrow immature natural killer cell
Liver natural killer cell
Limb Muscle T cell
Spleen B cell
Marrow immature B cell
Marrow macrophage
Lung T cell
Limb Muscle B cell
Fat T cell
Marrow late pro−B cell
Thymus DN1 thymic pro−T cell
Marrow immature T cell
Spleen T cell
Limb Muscle skeletal muscle satellite stem cell
Marrow B cell
Marrow immature NK T cell
Marrow pre−natural killer cell
Marrow Slamf1−negative multipotent progenitor cell
Marrow granulocyte monocyte progenitor cell
Marrow Slamf1−positive multipotent progenitor cell
Thymus immature T cell
Marrow megakaryocyte−erythroid progenitor cell
Marrow hematopoietic precursor cell
Marrow common lymphoid progenitor
Large Intestine enteroendocrine cell
Pancreas pancreatic PP cell
Lung epithelial cell of lung
Pancreas pancreatic A cell
Pancreas pancreatic D cell
Heart erythrocyte
Limb Muscle endothelial cell
Heart endothelial cell
Pancreas pancreatic ductal cell
Fat unknown cell type
Large Intestine enterocyte of epithelium of large intestine
Kidney kidney collecting duct epithelial cell
Brain Non−Myeloid neuron
Kidney epithelial cell of proximal tubule
Trachea mesenchymal cell
Fat endothelial cell
Trachea endothelial cell
Liver hepatocyte
Limb Muscle mesenchymal stem cell
Brain Non−Myeloid astrocyte
Brain Non−Myeloid endothelial cell
Lung ciliated columnar cell of tracheobronchial tree
Bladder bladder urothelial cell
Mammary Gland basal cell
Liver endothelial cell of hepatic sinusoid
Trachea blood cell
Trachea epithelial cell
Heart fibroblast
Pancreas pancreatic acinar cell
Skin epidermal cell
Mammary Gland endothelial cell
Heart cardiac muscle cell
Lung unknown cell type
Kidney endothelial cell
Lung lung endothelial cell
Lung stromal cell
Mammary Gland stromal cell
Brain Non−Myeloid Bergmann glial cell
Heart myofibroblast cell
Tongue keratinocyte
Pancreas pancreatic stellate cell
Skin basal cell of epidermis
Fat mesenchymal stem cell of adipose
Tongue basal cell of epidermis
Pancreas endothelial cell
Large Intestine Brush cell of epithelium proper of large intestine
Heart unknown cell type
Skin stem cell of epidermis
Mammary Gland luminal epithelial cell of mammary gland
Brain Non−Myeloid brain pericyte
Skin keratinocyte stem cell
Heart endocardial cell
Heart smooth muscle cell
Large Intestine epithelial cell of large intestine
Bladder bladder cell
Limb Muscle skeletal muscle satellite cell

**Supplementary Fig. 8: CELLECT-LDSC cellular associations with GrimAge (immune cells and precursors, non-immune cells)**

Results from CELLECT-LDSC cell-type enrichment analysis of GrimAge. Cell types are split into immune cells and immune cell precursors (top half of figure) and non-immune cells (bottom half of figure). Circles represent negative, log-transformed, unadjusted P values. Statistical analyses were conducted using one-sided t-tests. Black circles represent non-significant cell-trait associations at a FDR of 0.05. Black circles to the right of the dotted line represent nominally significant associations (i.e., cells with P value < 0.05 that fail to survive correction for multiple comparisons). Exact P values are contained in Supplementary Data 32.

CELLECT: CELL-type Expression-specific integration for Complex Traits; LDSC: LD Score Regression; SNP: single nucleotide polymorphism; FDR: false discovery rate.

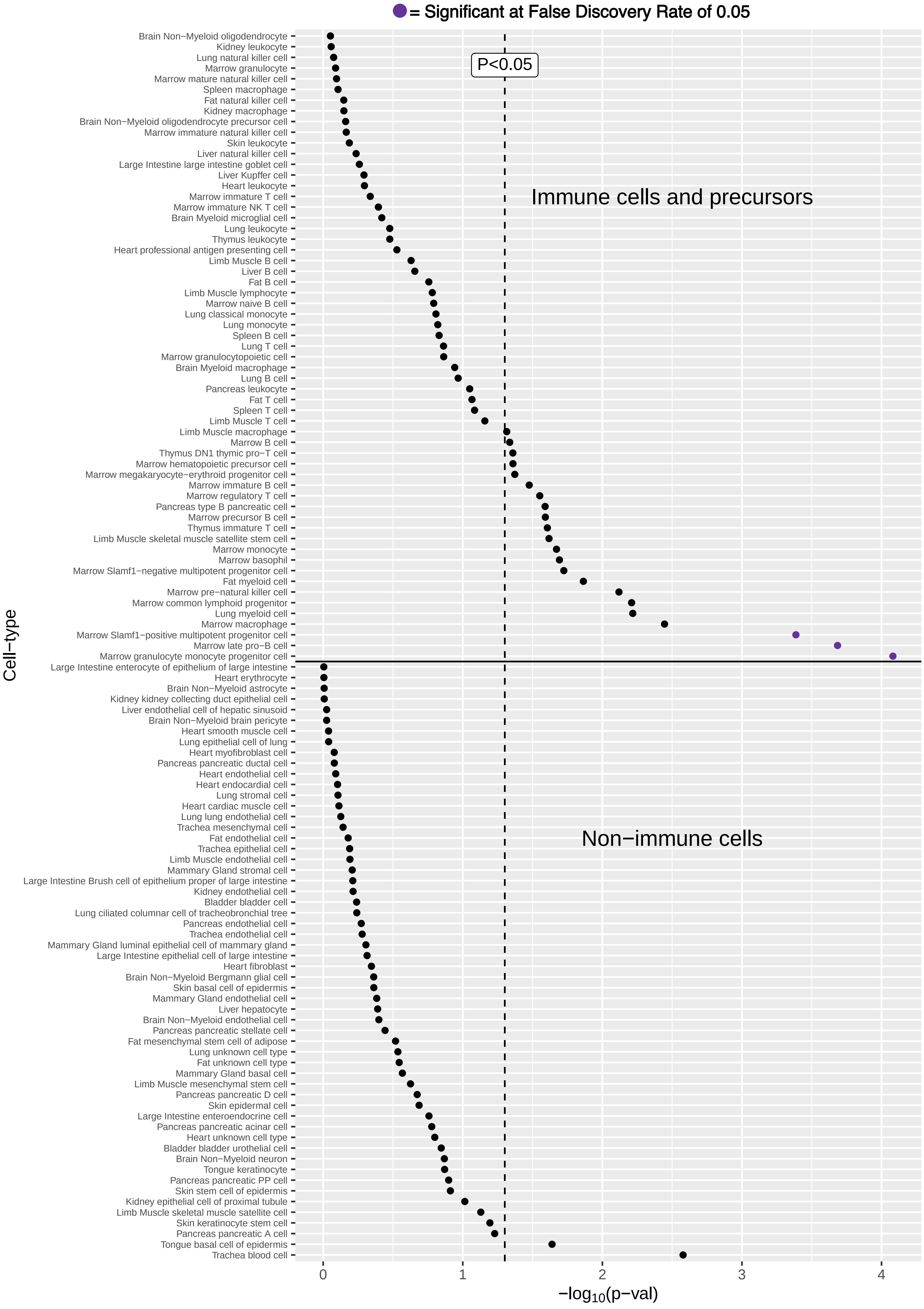
= Significant at False Discovery Rate of 0.05
P<0.05
Immune cells and precursors
Non−immune cells
Cell−type
−log10(p−val)
0
1
2
3
4
Brain Non−Myeloid oligodendrocyte
Kidney leukocyte
Lung natural killer cell
Marrow granulocyte
Marrow mature natural killer cell
Spleen macrophage
Fat natural killer cell
Kidney macrophage
Brain Non−Myeloid oligodendrocyte precursor cell
Marrow immature natural killer cell
Skin leukocyte
Liver natural killer cell
Large Intestine large intestine goblet cell
Liver Kupffer cell
Heart leukocyte
Marrow immature T cell
Marrow immature NK T cell
Brain Myeloid microglial cell
Lung leukocyte
Thymus leukocyte
Heart professional antigen presenting cell
Limb Muscle B cell
Liver B cell
Fat B cell
Limb Muscle lymphocyte
Marrow naive B cell
Lung classical monocyte
Lung monocyte
Spleen B cell
Lung T cell
Marrow granulocytopoietic cell
Brain Myeloid macrophage
Lung B cell
Pancreas leukocyte
Fat T cell
Spleen T cell
Limb Muscle T cell
Limb Muscle macrophage
Marrow B cell
Thymus DN1 thymic pro−T cell
Marrow hematopoietic precursor cell
Marrow megakaryocyte−erythroid progenitor cell
Marrow immature B cell
Marrow regulatory T cell
Pancreas type B pancreatic cell
Marrow precursor B cell
Thymus immature T cell
Limb Muscle skeletal muscle satellite stem cell
Marrow monocyte
Marrow basophil
Marrow Slamf1−negative multipotent progenitor cell
Fat myeloid cell
Marrow pre−natural killer cell
Marrow common lymphoid progenitor
Lung myeloid cell
Marrow macrophage
Marrow Slamf1−positive multipotent progenitor cell
Marrow late pro−B cell
Marrow granulocyte monocyte progenitor cell
Large Intestine enterocyte of epithelium of large intestine
Heart erythrocyte
Brain Non−Myeloid astrocyte
Kidney kidney collecting duct epithelial cell
Liver endothelial cell of hepatic sinusoid
Brain Non−Myeloid brain pericyte
Heart smooth muscle cell
Lung epithelial cell of lung
Heart myofibroblast cell
Pancreas pancreatic ductal cell
Heart endothelial cell
Heart endocardial cell
Lung stromal cell
Heart cardiac muscle cell
Lung lung endothelial cell
Trachea mesenchymal cell
Fat endothelial cell
Trachea epithelial cell
Limb Muscle endothelial cell
Mammary Gland stromal cell
Large Intestine Brush cell of epithelium proper of large intestine
Kidney endothelial cell
Bladder bladder cell
Lung ciliated columnar cell of tracheobronchial tree
Pancreas endothelial cell
Trachea endothelial cell
Mammary Gland luminal epithelial cell of mammary gland
Large Intestine epithelial cell of large intestine
Heart fibroblast
Brain Non−Myeloid Bergmann glial cell
Skin basal cell of epidermis
Mammary Gland endothelial cell
Liver hepatocyte
Brain Non−Myeloid endothelial cell
Pancreas pancreatic stellate cell
Fat mesenchymal stem cell of adipose
Lung unknown cell type
Fat unknown cell type
Mammary Gland basal cell
Limb Muscle mesenchymal stem cell
Pancreas pancreatic D cell
Skin epidermal cell
Large Intestine enteroendocrine cell
Pancreas pancreatic acinar cell
Heart unknown cell type
Bladder bladder urothelial cell
Brain Non−Myeloid neuron
Tongue keratinocyte
Pancreas pancreatic PP cell
Skin stem cell of epidermis
Kidney epithelial cell of proximal tubule
Limb Muscle skeletal muscle satellite cell
Skin keratinocyte stem cell
Pancreas pancreatic A cell
Tongue basal cell of epidermis
Trachea blood cell

**Supplementary Fig. 9: CELLECT-LDSC cellular associations with PhenoAge (immune cells and precursors, non-immune cells)**

Results from CELLECT-LDSC cell-type enrichment analysis of PhenoAge. Cell types are split into immune cells and immune cell precursors (top half of figure) and non-immune cells (bottom half of figure). Circles represent negative, log-transformed, unadjusted P values. Statistical analyses were conducted using one-sided t-tests. Purple circles represent cell types significantly enriched in PhenoAge-associated SNPs at a FDR of 0.05. Black circles represent non-significant associations. Black circles to the right of the dotted line represent nominally significant associations (i.e., cells with P value < 0.05 that fail to survive correction for multiple comparisons). Exact P values are contained in Supplementary Data 33.

CELLECT: CELL-type Expression-specific integration for Complex Traits; LDSC: LD Score Regression; SNP: single nucleotide polymorphism; FDR: false discovery rate.

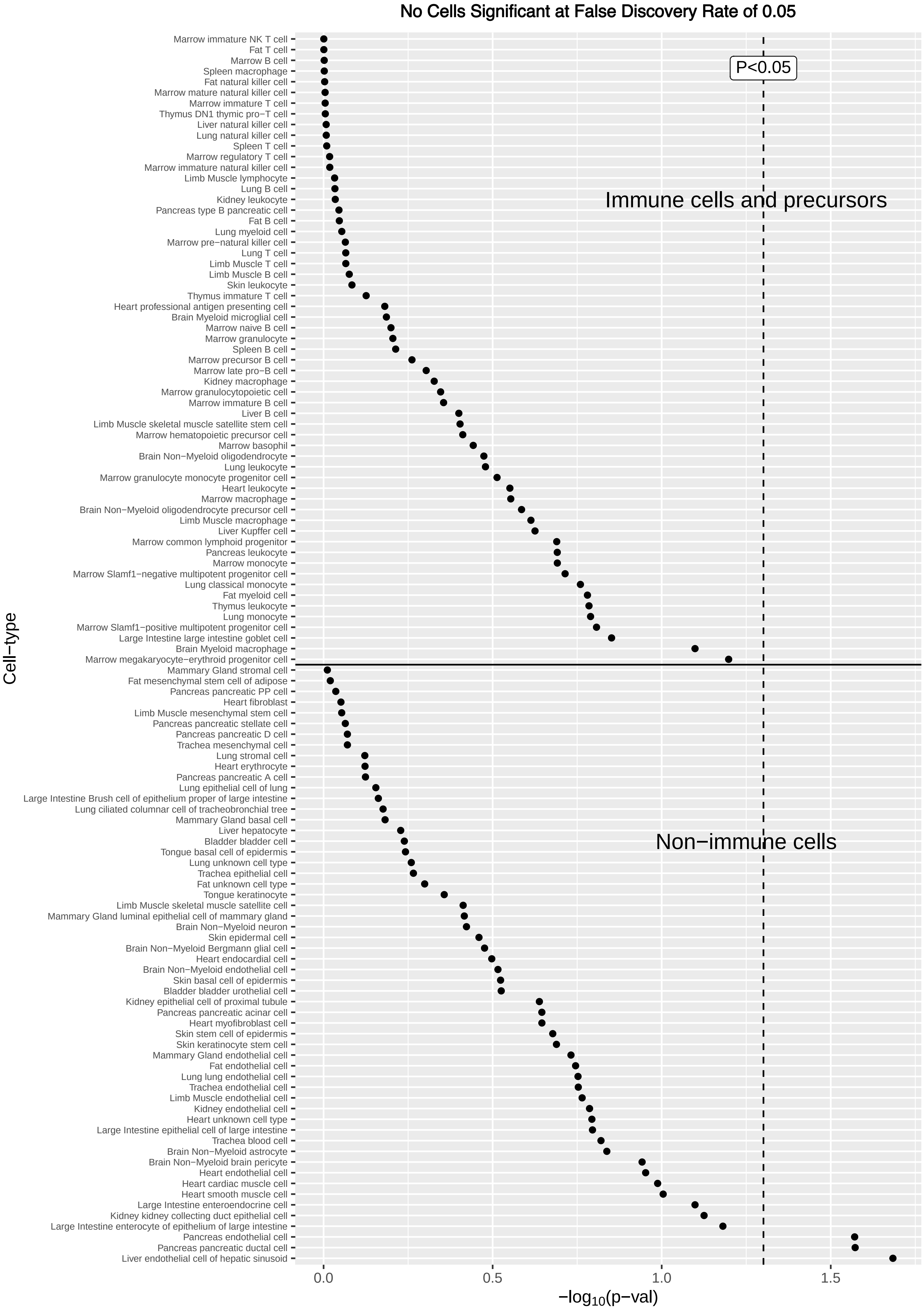
No Cells Significant at False Discovery Rate of 0.05
P<0.05
Immune cells and precursors
Non−immune cells
Cell−type
$-\log_{10}$(p−val)
Marrow immature NK T cell
Fat T cell
Marrow B cell
Spleen macrophage
Fat natural killer cell
Marrow mature natural killer cell
Marrow immature T cell
Thymus DN1 thymic pro−T cell
Liver natural killer cell
Lung natural killer cell
Spleen T cell
Marrow regulatory T cell
Marrow immature natural killer cell
Limb Muscle lymphocyte
Lung B cell
Kidney leukocyte
Pancreas type B pancreatic cell
Fat B cell
Lung myeloid cell
Marrow pre−natural killer cell
Lung T cell
Limb Muscle T cell
Limb Muscle B cell
Skin leukocyte
Thymus immature T cell
Heart professional antigen presenting cell
Brain Myeloid microglial cell
Marrow naive B cell
Marrow granulocyte
Spleen B cell
Marrow precursor B cell
Marrow late pro−B cell
Kidney macrophage
Marrow granulocytopoietic cell
Marrow immature B cell
Liver B cell
Limb Muscle skeletal muscle satellite stem cell
Marrow hematopoietic precursor cell
Marrow basophil
Brain Non−Myeloid oligodendrocyte
Lung leukocyte
Marrow granulocyte monocyte progenitor cell
Heart leukocyte
Marrow macrophage
Brain Non−Myeloid oligodendrocyte precursor cell
Limb Muscle macrophage
Liver Kupffer cell
Marrow common lymphoid progenitor
Pancreas leukocyte
Marrow monocyte
Marrow Slamf1−negative multipotent progenitor cell
Lung classical monocyte
Fat myeloid cell
Thymus leukocyte
Lung monocyte
Marrow Slamf1−positive multipotent progenitor cell
Large Intestine large intestine goblet cell
Brain Myeloid macrophage
Marrow megakaryocyte−erythroid progenitor cell
Mammary Gland stromal cell
Fat mesenchymal stem cell of adipose
Pancreas pancreatic PP cell
Heart fibroblast
Limb Muscle mesenchymal stem cell
Pancreas pancreatic stellate cell
Pancreas pancreatic D cell
Trachea mesenchymal cell
Lung stromal cell
Heart erythrocyte
Pancreas pancreatic A cell
Lung epithelial cell of lung
Large Intestine Brush cell of epithelium proper of large intestine
Lung ciliated columnar cell of tracheobronchial tree
Mammary Gland basal cell
Liver hepatocyte
Bladder bladder cell
Tongue basal cell of epidermis
Lung unknown cell type
Trachea epithelial cell
Fat unknown cell type
Tongue keratinocyte
Limb Muscle skeletal muscle satellite cell
Mammary Gland luminal epithelial cell of mammary gland
Brain Non−Myeloid neuron
Skin epidermal cell
Brain Non−Myeloid Bergmann glial cell
Heart endocardial cell
Brain Non−Myeloid endothelial cell
Skin basal cell of epidermis
Bladder bladder urothelial cell
Kidney epithelial cell of proximal tubule
Pancreas pancreatic acinar cell
Heart myofibroblast cell
Skin stem cell of epidermis
Skin keratinocyte stem cell
Mammary Gland endothelial cell
Fat endothelial cell
Lung lung endothelial cell
Trachea endothelial cell
Limb Muscle endothelial cell
Kidney endothelial cell
Heart unknown cell type
Large Intestine epithelial cell of large intestine
Trachea blood cell
Brain Non−Myeloid astrocyte
Brain Non−Myeloid brain pericyte
Heart endothelial cell
Heart cardiac muscle cell
Heart smooth muscle cell
Large Intestine enteroendocrine cell
Kidney kidney collecting duct epithelial cell
Large Intestine enterocyte of epithelium of large intestine
Pancreas endothelial cell
Pancreas pancreatic ductal cell
Liver endothelial cell of hepatic sinusoid
0.0
0.5
1.0
1.5

**Supplementary Fig. 10: CELLECT-LDSC cellular associations with multivariate longevity (immune cells and precursors, non-immune cells)**

Results from CELLECT-LDSC cell-type enrichment analysis of multivariate longevity. Cell types are split into immune cells and immune cell precursors (top half of figure) and non-immune cells (bottom half of figure). Circles represent negative, log-transformed, unadjusted P values. Statistical analyses were conducted using one-sided t-tests. Black circles represent non-significant cell-trait associations at a FDR of 0.05. Black circles to the right of the dotted line represent nominally significant associations (i.e., cells with P value < 0.05 that fail to survive correction for multiple comparisons). Exact P values are contained in Supplementary Data 34.

CELLECT: CELL-type Expression-specific integration for Complex Traits; LDSC: LD Score Regression; SNP: single nucleotide polymorphism; FDR: false discovery rate.
