# Supplementary material for: Multi-omic underpinnings of epigenetic aging and human longevity
Source: Nat Commun. 2023 Apr 19;14:2236. doi: 10.1038/s41467-023-37729-w (PMC10115892; doi:10.1038/s41467-023-37729-w)
Supplement: Supplementary file 3 — Description of Additional Supplementary Files [file 41467_2023_37729_MOESM3_ESM.pdf]

Description of Additional Supplementary Files

File Name: Supplementary Data 1
Description: Results of transcriptome-wide association study of intrinsic epigenetic age acceleration.

File Name: Supplementary Data 2
Description: Results of transcriptome-wide association study of HannumAge.

File Name: Supplementary Data 3
Description: Results of transcriptome-wide association study of GrimAge.

File Name: Supplementary Data 4
Description: Results of transcriptome-wide association study of PhenoAge.

File Name: Supplementary Data 5
Description: Results of transcriptome-wide association study of multivariate longevity.

File Name: Supplementary Data 6
Description: Conditionally significant transcriptome-wide association study findings.

File Name: Supplementary Data 7
Description: Transcriptome-wide association study findings with a FOCUS posterior inclusion probability > 0.5.

File Name: Supplementary Data 8
Description: PrismEXP results: Gene Ontology biological processes.

File Name: Supplementary Data 9
Description: PrismEXP results: Gene Ontology cellular components.

File Name: Supplementary Data 10
Description: PrismEXP results: Gene Ontology molecular functions.

File Name: Supplementary Data 11
Description: PrismEXP results: Gene Ontology gene sets linked to multiple aging traits.

File Name: Supplementary Data 12
Description: Drug target Mendelian randomization instrument SNP data.

File Name: Supplementary Data 13
Description: Results of drug-target Mendelian randomization analysis of intrinsic epigenetic age acceleration.

File Name: Supplementary Data 14
Description: Results of drug-target Mendelian randomization analysis of HannumAge.

File Name: Supplementary Data 15
Description: Results of drug-target Mendelian randomization analysis of GrimAge.

File Name: Supplementary Data 16
Description: Results of drug-target Mendelian randomization analysis of PhenoAge.

File Name: Supplementary Data 17
Description: Results of drug-target Mendelian randomization analysis of multivariate longevity.

File Name: Supplementary Data 18
Description: Results of phenome-wide association study of top results from intrinsic epigenetic age acceleration drug-target Mendelian randomization analysis.

File Name: Supplementary Data 19
Description: Results of phenome-wide association study of top results from HannumAge drug-target Mendelian randomization analysis.

File Name: Supplementary Data 20
Description: Results of phenome-wide association study of top results from GrimAge drug-target Mendelian randomization analysis.

File Name: Supplementary Data 21
Description: Results of phenome-wide association study of top results from PhenoAge drug-target Mendelian randomization analysis.

File Name: Supplementary Data 22
Description: Results of phenome-wide association study of top results from multivariate longevity drug-target Mendelian randomization analysis.

File Name: Supplementary Data 23
Description: Exposures assessed in metabolome-wide Mendelian randomization analysis.

File Name: Supplementary Data 24
Description: Metabolome-wide Mendelian randomization analysis instrument SNP data.

File Name: Supplementary Data 25
Description: Results of metabolome-wide Mendelian randomization analysis on intrinsic epigenetic age acceleration.

File Name: Supplementary Data 26
Description: Results of metabolome-wide Mendelian randomization analysis on PhenoAge.

File Name: Supplementary Data 27
Description: Results of metabolome-wide Mendelian randomization analysis on HannumAge.

File Name: Supplementary Data 28
Description: Results of metabolome-wide Mendelian randomization analysis on GrimAge.

File Name: Supplementary Data 29
Description: Results of metabolome-wide Mendelian randomization analysis on multivariate longevity.

File Name: Supplementary Data 30
Description: Results of CELLECT cell-type enrichment analysis of intrinsic epigenetic age acceleration.

File Name: Supplementary Data 31
Description: Results of CELLECT cell-type enrichment analysis of HannumAge.

File Name: Supplementary Data 32
Description: Results of CELLECT cell-type enrichment analysis of GrimAge.

File Name: Supplementary Data 33
Description: Results of CELLECT cell-type enrichment analysis of PhenoAge.

File Name: Supplementary Data 34
Description: Results of CELLECT cell-type enrichment analysis of multivariate longevity.

File Name: Supplementary Data 35
Description: Immune trait exposures assessed in Mendelian randomization analysis of immune traits on aging traits.

File Name: Supplementary Data 36
Description: Mendelian randomization analysis of immune traits on aging traits instrument SNP data.

File Name: Supplementary Data 37
Description: Results of Mendelian randomization analysis of immune traits on multivariate longevity.

File Name: Supplementary Data 38
Description: Results of Mendelian randomization analysis of immune traits on intrinsic epigenetic age acceleration.

File Name: Supplementary Data 39
Description: Results of Mendelian randomization analysis of immune traits on HannumAge.

File Name: Supplementary Data 40
Description: Results of Mendelian randomization analysis of immune traits on GrimAge.

File Name: Supplementary Data 41
Description: Results of Mendelian randomization analysis of immune traits on PhenoAge.

File Name: Supplementary Data 42
Description: Mendelian randomization analysis of aging traits on immune traits instrument SNP data.

File Name: Supplementary Data 43
Description: Results of Mendelian randomization analysis of intrinsic epigenetic age acceleration on immune traits.

File Name: Supplementary Data 44
Description: Results of Mendelian randomization analysis of HannumAge on immune traits.

File Name: Supplementary Data 45
Description: Results of Mendelian randomization analysis of GrimAge on immune traits.

File Name: Supplementary Data 46
Description: Results of Mendelian randomization analysis of PhenoAge on immune traits.

File Name: Supplementary Data 47
Description: Results of Mendelian randomization analysis of multivariate longevity on immune traits.
