# Supplementary material for: Multi-omic underpinnings of epigenetic aging and human longevity
Source: Nat Commun. 2023 Apr 19;14:2236. doi: 10.1038/s41467-023-37729-w (PMC10115892; doi:10.1038/s41467-023-37729-w)
Supplement: Supplementary file 5 — Reporting Summary [file 41467_2023_37729_MOESM5_ESM.pdf]

Corresponding author(s): Falk W. Lohoff

Last updated by author(s): 03/17/2023

Reporting Summary

Nature Portfolio wishes to improve the reproducibility of the work that we publish. This form provides structure for consistency and transparency in reporting. For further information on Nature Portfolio policies, see our Editorial Policies and the Editorial Policy Checklist.

Statistics

For all statistical analyses, confirm that the following items are present in the figure legend, table legend, main text, or Methods section.

n/a	Confirmed	
☐	☒	The exact sample size (*n*) for each experimental group/condition, given as a discrete number and unit of measurement
☐	☒	A statement on whether measurements were taken from distinct samples or whether the same sample was measured repeatedly
☐	☒	The statistical test(s) used AND whether they are one- or two-sided<br>*Only common tests should be described solely by name; describe more complex techniques in the Methods section.*
☒	☐	A description of all covariates tested
☐	☒	A description of any assumptions or corrections, such as tests of normality and adjustment for multiple comparisons
☐	☒	A full description of the statistical parameters including central tendency (e.g. means) or other basic estimates (e.g. regression coefficient) AND variation (e.g. standard deviation) or associated estimates of uncertainty (e.g. confidence intervals)
☐	☒	For null hypothesis testing, the test statistic (e.g. *F*, *t*, *r*) with confidence intervals, effect sizes, degrees of freedom and *P* value noted<br>*Give P values as exact values whenever suitable.*
☒	☐	For Bayesian analysis, information on the choice of priors and Markov chain Monte Carlo settings
☒	☐	For hierarchical and complex designs, identification of the appropriate level for tests and full reporting of outcomes
☐	☒	Estimates of effect sizes (e.g. Cohen's *d*, Pearson's *r*), indicating how they were calculated

Our web collection on statistics for biologists contains articles on many of the points above.

Software and code

Policy information about availability of computer code

Data collection

No software was used to collect data for this study. All analyses relied entirely on publicly available data (see "Data").

Data analysis

The softwares used in this study are available at the following online repositories. R package TwoSampleMR version 0.5.6: https://mrcieu.github.io/TwoSampleMR/; R package MendelianRandomization version 0.6.0: https://cran.r-project.org/web/packages/MendelianRandomization/index.html/; R package ggforestplot version 0.1.0 nmr-data-analysis-tutorial.Rmd: https://github.com/nightingalehealth/ggforestplot/blob/master/vignettes/nmr-data-analysis-tutorial.Rmd; Python package LDSC version 1.0.1 (https://github.com/bulik/ldsc); R FUSION pipeline, March 16th, 2020 version: http://gusevlab.org/projects/fusion/; Python package FOCUS version 0.6.10 (https://github.com/bogdanlab/focus); R package coloc version 5.1.0.1: https://cran.r-project.org/web/packages/coloc/index.html; Python package PrismEXP version 1.86: https://github.com/MaayanLab/prismexp; Python package CELLECT version 1.3.0: https://github.com/perslab/CELLECT; Python package CELLEX version 1.2.1: https://github.com/perslab/CELLEX.

Fig. 1 was made using BioRender.com. Fig. 2 was made using R package TWAS Plotter version 1.0: (https://github.com/opain/TWAS-plotter) and R package ggven version 0.1.8: (https://github.com/yanlinlin82/ggvenn). Fig. 3 and Supplementary Figs. 1-10 were made using R package EnhancedVolcano version 1.16.0: https://github.com/kevinblighe/EnhancedVolcano. Fig. 4 was made using R package ggplot2 version 3.3.5: https://cloud.r-project.org/web/packages/ggplot2/index.html.

R version 4.2.1 was used to format data for analyses.

For manuscripts utilizing custom algorithms or software that are central to the research but not yet described in published literature, software must be made available to editors and reviewers. We strongly encourage code deposition in a community repository (e.g. GitHub). See the Nature Portfolio guidelines for submitting code & software for further information.

Data

Policy information about availability of data

All manuscripts must include a data availability statement. This statement should provide the following information, where applicable:

- Accession codes, unique identifiers, or web links for publicly available datasets
- A description of any restrictions on data availability
- For clinical datasets or third party data, please ensure that the statement adheres to our policy

All analyses in this study were conducted using publicly available data. URLs for the source datasets are as follows: epigenetic age acceleration GWAS summary statistics: https://datashare.ed.ac.uk/handle/10283/3645; multivariate longevity GWAS summary statistics: https://datashare.ed.ac.uk/handle/10283/3599; sCCA weights (used for transcriptomic imputation) and 1000 Genomes Project Phase 3 European genomic reference data (used for transcriptomic imputation and MR): http://gusevlab.org/projects/fusion/; eQTLgen whole blood eQTL data used for MR of the druggable genome: https://www.eqtlgen.org/; Nightingale metabolomics GWAS summary statistics used for MR: https://gwas.mrcieu.ac.uk/, batch: met-d; Immune cell trait GWAS summary statistics used for MR: https://gwas.mrcieu.ac.uk/, ebi-a-90001391 through ebi-a-90002121; scRNA-seq data used for cell-type enrichment analysis: https://tabula-muris.ds.czbiohub.org/; FinnGen R5 data used for PheWAS analyses: https://r5.finngen.fi/; 2021 GO biological processes, molecular functions, and cellular components gene sets used for PrismEXP analyses: https://maayanlab.cloud/Enrichr/#libraries.
All data generated in this study upon which conclusions are based are available in the Supplementary Data.
Source data for Figs. 2-4 and Supplementary Figs. 1-10 are provided with this paper.

Human research participants

Policy information about studies involving human research participants and Sex and Gender in Research.

Reporting on sex and gender	All analyses in this study were conducted using publicly available genome-wide association study data (GWAS). Therefore, sex and gender were not considered in design of this study. Additionally, disaggregated sex and gender data was not generated because sex-stratified genome-wide associations studies of multivariate longevity and epigenetic age acceleration were not available. The original GWAS summary statistics were generated using additive models that accounted for covariates including self-reported sex. See the links above for more information regarding the statistical analysis of the original GWAS data.
Population characteristics	This study uses only GWAS summary-level data (i.e., this study does not used individual-level data). See the GWAS study links provided in the "Data" section of the Reporting Summary for information regarding population characteristics for the participating cohorts in the original studies.
Recruitment	This study uses only GWAS summary-level data (i.e., this study does not used individual-level data). See the GWAS study links provided in the "Data" section of the Reporting Summary for information regarding recruitment of the participating cohorts in the original studies.
Ethics oversight	This study uses only publicly available GWAS summary-level data. The original GWAS studies each had approval from their respective review boards and ethics oversight authorities.

Note that full information on the approval of the study protocol must also be provided in the manuscript.

Field-specific reporting

Please select the one below that is the best fit for your research. If you are not sure, read the appropriate sections before making your selection.

- [x] Life sciences
- [ ] Behavioural & social sciences
- [ ] Ecological, evolutionary & environmental sciences

For a reference copy of the document with all sections, see nature.com/documents/nr-reporting-summary-flat.pdf

Life sciences study design

All studies must disclose on these points even when the disclosure is negative.

Sample size	Because statistical power for transcriptomic imputation and Mendelian randomization analyses are dependent upon sample size, we maximized power of these analyses by including the largest GWAS data available for each exposure and outcome. See Methods for sample sizes for all GWAS cohorts used in this study.
Data exclusions	We used single nucleotide polymorphisms associated with the exposures in each of our analyses (i.e., gene expression in the transcriptomic imputation and drug-target Mendelian randomization analyses, circulating metabolites in the metabolome-wide MR, and immune cell components in the downstream MR analysis). For the transciptomic imputation and drug-target MR analyses, we only used genetic variants located within or near the genomic loci of the genes included in the analyses. Genetic variants were conditionally independent and associated with gene expression at a specified, analysis-dependent P value threshold (i.e., default association criteria for the FUSION TWAS and P-value < Sxl0-8 for the MR analyses). For the other MR analyses, we used standard MR genetic instrument selection criteria (P-value < Sxl0-8) and selected genetic variants associated with each exposure irregardless of genomic position as is conventional in polygenic MR analysis. We

	excluded variants that were found in the exposure phenotype but not the outcome phenotype from our analyses. Additionally, when specified in Methods, we excluded variants that explained more variation in the outcome phenotype than in the exposure phenotype from our MR analyses so as to minimize the influence of reverse causality on our findings.
Replication	Due to a lack of independent GWAS data on the phenotype analyzed in this study, we did not perform independent replication. However, many of our transcriptomic findings reflected previously identified GWAS findings. Moreover, we used multiple methods to interrogate similar questions in this study (e.g., transcriptomic imputation and drug-target MR, cell-type enrichment analysis and MR of immune cell components on aging outcomes). The results of these analyses converged to some extent, as described in Results.
Randomization	Transcriptomic imputation and Mendelian randomization rely on genetic variation, which is randomized at conception for each study participant (i.e., genetic alleles are independently allocated at this time).
Blinding	The design of this study did not include the assignment of participants to groups. Therefore, blinding in the conventional sense is not applicable. GWAS participants and those performing phenotypic assessments were very likely unaware of participants' genotypes, thus minimizing any possibility of participant or observer bias in the source GWASs.

Reporting for specific materials, systems and methods

We require information from authors about some types of materials, experimental systems and methods used in many studies. Here, indicate whether each material, system or method listed is relevant to your study. If you are not sure if a list item applies to your research, read the appropriate section before selecting a response.

Materials & experimental systems

n/a	Involved in the study
☒	☐ Antibodies
☒	☐ Eukaryotic cell lines
☒	☐ Palaeontology and archaeology
☒	☐ Animals and other organisms
☒	☐ Clinical data
☒	☐ Dual use research of concern

Methods

n/a	Involved in the study
☒	☐ ChIP-seq
☒	☐ Flow cytometry
☒	☐ MRI-based neuroimaging
